# An Evaluation of Neurotoxicity Following Fluoride Exposure from Gestational Through Adult Ages in Long-Evans Hooded Rats

**DOI:** 10.1007/s12640-018-9870-x

**Published:** 2018-02-05

**Authors:** Christopher A. McPherson, Guozhu Zhang, Richard Gilliam, Sukhdev S. Brar, Ralph Wilson, Amy Brix, Catherine Picut, G. Jean Harry

**Affiliations:** 1 Neurotoxicology Group, National Toxicology Program Laboratory, National Institute of Environmental Health Sciences, Mail Drop C1-04, P.O. Box 12233, Research Triangle Park, NC 27709 USA; 2 Social & Scientific Systems, Inc, Durham, NC 27703 USA; 3 Cellular & Molecular Pathology Branch, Division of the National Toxicology Program, NIEHS, Research Tringle Park, NC 27709 USA; 4 Experimental Pathology Laboratories, Research Triangle Park, NC USA; 5 Charles River Laboratories, Inc, Durham, NC USA

**Keywords:** Fluoride, Learning and memory, Avoidance, Activity, Neurodevelopment

## Abstract

At elevated levels, fluoride (F^−^) exposure has been associated with adverse human health effects. In rodents, F^−^ exposure has been reported to induce deficits in motor performance and learning and memory. In this study, we examined Long-Evans hooded male rats maintained on a standard diet (20.5 ppm F^−^) or a low F^−^ diet (3.24 ppm F^−^) with drinking water exposure to 0, 10, or 20 ppm F^−^ from gestational day 6 through adulthood. At postnatal day 25, brain F^−^ levels were 0.048 or 0.081 μg/g and femur 235 or 379.8 μg/g for 10 and 20 ppm F^−^, respectively. Levels increase with age and in adults, levels for plasma were 0.036 or 0.025 μg/ml; for the brain 0.266 or 0.850 μg/g; and for the femur, 681.2 or 993.4 μg/g. At these exposure levels, we observed no exposure-related differences in motor, sensory, or learning and memory performance on running wheel, open-field activity, light/dark place preference, elevated plus maze, pre-pulse startle inhibition, passive avoidance, hot-plate latency, Morris water maze acquisition, probe test, reversal learning, and Y-maze. Serum triiodothyronine (T3), thyroxine (T4), and thyroid stimulating hormone (TSH) levels were not altered as a function of 10 or 20 ppm F^−^ in the drinking water. No exposure-related pathology was observed in the heart, liver, kidney, testes, seminal vesicles, or epididymides. Mild inflammation in the prostate gland was observed at 20 ppm F^−^. No evidence of neuronal death or glial activation was observed in the hippocampus at 20 ppm F^−^.

**Electronic supplementary material:**

The online version of this article (10.1007/s12640-018-9870-x) contains supplementary material, which is available to authorized users.

## Introduction

Many US public water systems add fluoride to finished water as a preventive against dental caries with a US Public Health Service recommended water fluoridation level of 0.7 ppm. Under the Safe Drinking Water Act, the US Environmental Protection Agency (EPA) set the current enforceable upper concentration limit at 4 ppm in drinking water derived from sources with naturally high fluoride to prevent skeletal fluorosis. The secondary drinking water standard is 2 ppm and systems distributing water around this level are required to notify the affected public (US EPA 2013). The EPA proposed a reference dose of 0.08 mg/kg/day for protection against dental fluorosis and skeletal fractures (US EPA 2010). This is equivalent to a daily dose of ~ 5.6 mg for an adult and ~ 1.6 mg for a child. Studies examining associations between drinking water fluoride and neurological deficits or dysfunction have generated concerns for human exposure. Studies of sample populations in fluoride-endemic areas suggested an inverse association between high levels of naturally occurring fluoride in water and intelligence quotient; however, the level of confidence in these conclusions is low (Choi et al. 2012; Sutton et al. 2015). A recent study by Bashash et al. (2017) reported an association between prenatal maternal urine fluoride levels (0.90 ± 0.35 ppm) and performance of offspring (6–12 years of age) on tests of intelligence.

The question of potential neurological effects from fluoride exposures in experimental rodent models was examined in a recent systematic review of behavior (NTP (National Toxicology Program) 2016). Translation of findings from these studies to human exposures is often difficult because of the high levels of fluoride (F^−^, > 100 ppm) administered or routes of exposure not representative of drinking water. The majority of rat studies have focused on exposure to adult males with only a limited number examining developmental exposure (Wang et al. 2004; Niu et al. 2009; El-lethey et al. 2010; Banji et al. 2013; Jiang et al. 2014b; Wei et al. 2014). At drinking water concentrations of ≥ 100 ppm F^−^, various studies suggested performance deficits in rats on learning and memory tasks (Basha et al. 2011; Basha and Sujitha 2012; Jiang et al. 2014a; Zhu et al. 2017). However, many of these findings occurred in the presence of potential motor dysfunction or general toxicity (Bera et al. 2007; Basha and Sujitha 2012; Balaji et al. 2015); thus, diminishing confidence in any conclusion of learning deficits. Studies examining F^−^ exposures ≤ 100 ppm present an inconsistent pattern of neurobehavioral effects. A number of studies reported effects on motor or learning performance in rats at drinking water concentrations of ~ 2 to 50 ppm F^−^ (Bera et al. 2007; Gao et al. 2008; Liu et al. 2009; El-lethey et al. 2010; Jiang et al. 2014b; Wang et al. 2004; Wu et al. 2006; Wei et al. 2014; Bartos et al. 2015; Dong et al. 2015a). Many of these studies had limitations in experimental design and often utilized latency measures of learning that were dependent on a motor response (NTP 2016). Rather than examining improvement in performance with training (acquisition), many studies relied on a single assessment of learning and a single latency measure at the end of training (Gao et al. 2008, 2009a, b; Jiang et al. 2014b; Wei et al. 2014; Dong et al. 2015a), not taking into consideration the initial latency differences. In studies that reported acquisition data, longer latencies were often observed; yet, performance improved with training (Gui et al. 2010; Basha et al. 2011; Basha and Sujitha 2012; Zhu et al. 2017). These findings suggested a need to discriminate between effects on motor and learning in assessing F^−^ neurotoxicity.

The current study was designed to address issues identified in the NTP systematic review (NTP 2016) of determining low to moderate levels of evidence for effects of F^−^ exposure on learning and memory and to address the paucity of quality studies conducted at exposure levels near the recommended level for community water fluoridation in the USA. Equivalent human daily water intakes of 1.74 mg F/day for an adult or 0.63–1.23 mg/day for 1 to 14 years of age (US EPA 2010) have been approximated in rodents using drinking water concentrations of 7 to 9 ppm F^−^ (NTP 2016). In the current study, the top dose of 20 ppm F^−^ was selected based upon the US Environmental Protection Agency’s Maximum Contaminant Level of 4 ppm and the conventional wisdom that a 5-fold increase in dose is required to achieve comparable human serum levels (Dunipace et al. 1995; NRC 2006). We examined the effects of F^−^ exposure initiated during prenatal development by replicating and refining testing paradigms, assessing multiple sensory and motor modalities, and examining learning and memory across different test paradigms. Additional effects reported for F^−^ exposure that may influence behavior were examined (i.e., the thyroid hormone levels, kidney, liver, reproductive system histopathology, and neuronal and glia morphology in the hippocampus) to obtain a better understanding of observed effects.

## Materials and Methods

### Animals and Dosing

Timed-pregnant Long-Evans hooded rats (Charles River Laboratory, Raleigh, NC) were obtained (four shipments over 6 months) on gestational day (GD) 4 and individually housed in ventilated cages (Techniplast, West Chester, PA) with autoclaved, hardwood bedding (PJ Murphy, Montville, NY) within a semi-barrier room (40–60% humidity; 12-h light/dark cycle, 6:00–18:00; 20–24 °C). Dams were randomly assigned to exposure group. Two drinking water control groups were maintained on reverse osmosis drinking water (RO-H_2_O). One group (G1) was maintained on a standard rodent chow (Teklad 2918; Envigo, Madison, WI) and the second (G2) was maintained on a low-fluoride chow (Teklad Custom Diet TD.160173). To examine the effect of fluoride in the drinking water while controlling for dietary fluoride, rats were exposed to the low-fluoride chow and water supplemented with either 10 ppm F^−^ (G3) or 20 ppm F^−^ (G4). Dosing solutions were prepared fresh weekly with sodium fluoride (NaF; lot no. X0044851; 99.9%; Materion, Milwaukee, WI). Fluoride levels in drinking water were confirmed [RO-H_2_O, < 0.2 ppm; 10 and 20 ppm F^−^within ≤ 5% of target] (analytical method: EPA 300.0; Pace Analytical, Huntersville, NC). The Teklad 2918 diet contained 20.5 ppm F^−^ and the TD.160173 custom diet contained 3.24 ppm F^−^ (Official Methods of Analysis Methods 944.08 and 978.03; AOAC International, Gaithersburg, MD; Covance, Madison, WI). Food and water were available ad libitum. Exposure to the dams began on GD6 and continued throughout lactation. Pups were allowed free access to drinking water, beginning consumption around PND14, and continued on the same level of exposure after weaning until study termination. All animal procedures were conducted in accordance with protocols approved by the NIEHS Animal Care and Use Committee within AAALAC approved animal facilities.

The day of birth, postnatal day (PND) 0, occurred within 12 h with similar pup numbers across groups. On PND4, pups within each group were cross-fostered to establish litters of ten pups (six male and four female pups). Male pups were toe-tattooed (BD PrecisionGlide 27G needle) and randomly assigned to four behavioral testing groups (cohorts) ensuring only one male pup per gestational and postnatal litter assigned for any one endpoint. [Cohort 1: running wheel (RW; PND24), elevated plus maze (EPM; PND30), passive avoidance (PA; PND55), hot plate; Cohort 2: EPM (PND29); Y-maze (PND38); Cohort 3: motor activity (MA; PND40), light/dark place preference (L/D; PND43), Morris water maze (MWM; PND60); Cohort 4: pre-pulse startle inhibition (PPI; PND61–63), adult EPM (PND70)]. Excess male pups were identified as unassigned. Female pups were not used. Age of eye-opening for both eyes was similar across groups. Male pups weaned on PND21 were group-housed 2–3 per cage depending on the final terminal age on study to adhere to housing guidelines. When examined as adults, (> PND90), rats in the 20 ppm F^−^ dose group showed evidence of mild fluorosis (Supplementary Fig. S1) similar to that reported by Catani et al. (2010) after 78 days of 25 ppm F^−^ exposure.

### Behavioral Testing

Handling of rats for behavioral testing followed NTP guidelines for neurobehavioral testing (NTP 2015). All testing was conducted between 10:00–15:00 h. Assignment of rats to testing apparatus and time of testing was counterbalanced. Body weights were similar across groups prior to the start of testing for PA, PPI, or MWM (Supplementary Table S1). Accurate camera tracking by Ethovision XT 11.5 (Noldus, Wageningen, the Netherlands) was confirmed for each rat and incorrect points were edited and corrected per Noldus manual.

#### Running Wheel Activity

PND24 rats were transferred to testing room and allowed to acclimate under identical housing conditions for 24 h. Individual rats were transferred to a filter-top cage (396 × 215 × 172 mm) with a stainless steel RW (Mini-Mitter®; Respironics Co., Bend, OR) that limited light levels to 91 and 8 lm for light/dark periods, respectively. Computer-assisted recording of wheel revolutions in 15-min epochs (Vital View Data Acquisition, Respironics Co.) were collected over dark (18:00–6:00 EST) and light (6:00–18:00 EST) phases. Original food and water exposure was maintained. A linear mixed-effects repeated measures ANOVA (RM ANOVA) was used to analyze total daily RW rotations for the dark and light phases.

#### Elevated Plus Maze

To assess exploratory activity and anxiety-related performance (Walf and Frye 2007), naïve rats (PND30), rats with prior RW experience (PND31), and a separate group of naïve adult rats (PND72) were assessed in the EPM (a dark plexiglass apparatus: central area (10 × 10 cm), two opposing open arms (50 × 10 cm), two opposing enclosed arms (50 × 10 × 40cm)) elevated 50 cm above a dark floor. Each rat was placed in the central area facing an open arm and allowed 7 min to freely explore. Based on previous reports of a diminished EPM “cautious response” in fluoride exposed rats (Bartos et al. 2015), an additional testing paradigm was used to examine PND30 rats of G2 and G4. The paradigm was modified to include a linear-shadow cast over the maze as representative of a predator. Behavior was video-captured (Ethovision XT 11.5 Applications Manual) and the number of entries, percent time spent, and total distance traveled in arms were recorded. Wilcoxon Rank Sum tests were used to analyze number of entries and duration. Student’s *t* tests were used to analyze distance traveled.

#### Locomotor Activity

On PND40, exploratory motor activity was measured in an open field chamber (42 × 42 cm; Columbus Instruments, Columbus, OH) outfitted with photocell detectors (0.32 cm diameter) spaced 5 cm from floor and 1.27 cm linearly apart around the chamber. Ambulatory activity was recorded in 5-min epochs over a 45-min test session. Total ambulatory activity, time spent in the margin (one-photocell width from the wall), and activity within the center area (20 × 20 cm) were recorded. Student’s *t* tests were used to analyze total ambulatory activity, total ambulatory time, total distance traveled, and ambulatory activity acclimation. Wilcoxon Rank Sum tests were used to analyze activity total margin time, margin distance, and center distance traveled. RM ANOVA was used to analyze ambulatory activity in 5-min epochs.

#### Light/Dark Place Preference

To examine exploratory activity and preference for the dark chamber, a PND43 rat was placed in the lighted side of a 2-sided plexiglass chamber (68 × 21 × 34 cm; with a clear and a dark chamber, 34 × 21 × 34 cm). For 5 min, entries into and total time spend in lighted side were video-captured (Ethovision XT) and analyzed by Wilcoxon Rank Sum tests.

#### Passive Avoidance

The ability of a rat to learn to withhold a normally preferred response was assessed using a Gemini Avoidance System (San Diego Instruments, San Diego, CA). On PND55, a rat was placed into the start chamber modified by a white covering on back and side walls with the gate closed. After 120 s, the house light and cue light were turned on and the gate raised. Upon crossing to the dark side, the gate closed and a 3-s 0.5-mA floor-grid shock was delivered. The rat was removed after 10 s. This sequence was repeated every 24 h. Response latency was recorded with a maximum of 300 s. RM ANOVA was used to analyze latency over sessions, excluding day 1. The relative change from the first trial to the last trial was analyzed by Kruskal-Wallis rank sum test and Wilcoxon rank sum test. The percent reaching maximum was analyzed by Fisher’s exact test.

#### Hot-Plate Latency

Forty-eight hours after cessation of PA, pain threshold was determined as the latency to respond (jump or link of hindpaw; 2-min cutoff) to being placed on a 55 °C hot-plate platform (IITC Life Science, Woodland Hills, CA). Latency was analyzed by Student’s *t* test.

#### Startle Response and Pre-Pulse Startle Inhibition

PND 61–62 rats were assessed for auditory startle response, habituation, and PPI as a measure of sensorimotor gating using a computer-assisted SR-LAB startle apparatus (San Diego Instruments). Background noise level was set at 65 dB. Following a 5-min habituation period, the session began with a 120-dB trial, followed by 5120 dB trials; 2 blocks of 31 trials [2 no-stimulus trials, 6 acoustic startle stimuli (40-msec null period followed by 40-msec 120 dB pulse) trials alone, 18 pre-pulse stimulus trials (40-msec null period followed by 20-msec pre-pulse of 68, 71, 77, and 80 dB followed by a 100-msec null period and a 40-msec 120-dB pulse; for an entire recording period of 200 msec) presented in a random order, followed by 120-dB trials. Trials were presented at 15 s variable inter-trial intervals (ITI; 5–25 s). Habituation was calculated as difference between first and the last block of 120-dB trials. Pre-pulse startle inhibition was calculated as a percentage of the median 120 dB startle response. Wilcoxon Rank Sum tests were used to analyze the first 120 dB (Vmax) responses and startle habituation. RM ANOVA was used to analyze 120-dB startle responses (Vmax). Pre-pulse startle inhibition was analyzed by two-way ANOVA with dose and pre-pulse type as factors. Negative PPI values were set to 0.

#### Morris Water Maze

**Fig. 1:**
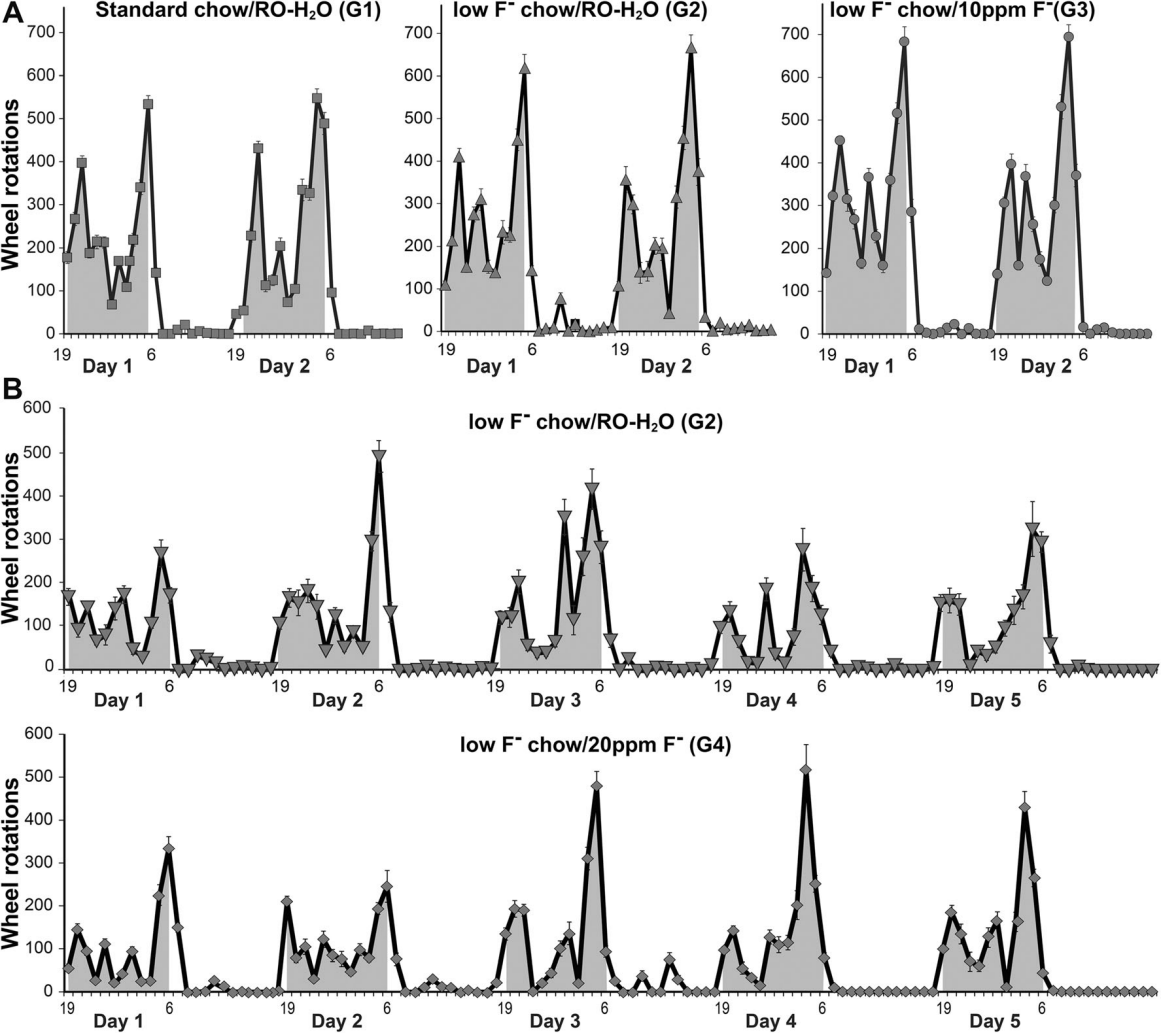
Running Wheel activity in postnatal day (PND) 25 Long-Evans hooded male rats. (**a**) Mean hourly wheel rotations during light and dark phases over 2 days. G1: standard chow/RO-H_2_O (*n* = 15); G2: low-F^−^ chow/RO-H_2_O (*n* = 15); and G3: low-F^−^ chow/10 ppm F^−^ drinking water (*n* = 16). Drinking water exposure began on gestational day 6. (**b**) Mean hourly wheel rotations over 5 days (mean ± SEM) for G2 (*n* = 10) and G4: low-F^−^ chow/20 ppm F^−^ drinking water (*n* = 10). Shaded areas represent dark phase of elevated activity

PND60 rats were transferred to the testing room 24 h prior to testing and maintained under normal home-cage conditions. A circular plastic tank (183 × 62 cm) filled with opaque water to 52 cm (10 cm distance from the water surface to the lip of tank) was allowed to equilibrate to room temperature (25 °C). Water was changed every 2 days. For cued learning, days 1 and 2, white curtains surrounded the tank (NTP 2015) and a clear visible plexiglass platform (10-cm diameter; Stoelting; Wood Dale, IL) was placed within one quadrant of the pool, 36 cm from the tank wall and raised 1.5 cm above the water and identified with a flag. The rat was placed into the pool in the quadrant opposite the platform, facing the wall of the tank and allowed 90 s to find the platform. The rat remained on the platform for 20 s before removal. Three trials, 10 min ITI, were run daily for 2 days. The platform location changed daily. For spatial learning, four geometrical figures (1 × 1 m) were placed on curtain walls at tank rim level as cues. The platform was submerged (1.5 cm) in a quadrant different from cued learning. Rats were placed in the tank within a pre-assigned quadrant and allowed 90 s to find platform. Three daily trials, alternating start location, were administered (10-min ITI). Rats were trained seven consecutive days, reaching the criteria of > 85% of control animals showing a > 50% decrease from original latency. A probe test was conducted 24 h following the final training trial to examine reference memory. The platform was removed and rats placed within quadrant opposite to goal quadrant (GQ) for 90 s. Reversal learning was initiated 48 h later. With the platform in a new quadrant, hidden platform training was conducted (three trials/day; 4 days). A probe test was conducted 24 h later. Video-captured images were analyzed using Ethovision XT. Acquisition was measured by latency to reach the platform zone (PZ; expanded to 15 cm (Blokland et al. 2004; Vorhees and Williams 2006). In the probe tests, latency to the first entry into GQ or PZ was recorded. The number of visits to the PZ, number of visits to each quadrant, and total time in each quadrant were recorded for each 30-s epoch. Wilcoxon Rank Sum tests were used to analyze latency on the first trial of cued learning. For each day, mean daily latencies were calculated and analyzed within each test phase of the MWM by RM ANOVAs employing a mixed model with autoregressive (1) (AR(1)) covariance due to the correlation between days. Comparisons across dose groups for any 1 day were analyzed by ANOVA. In the hidden platform sessions, the percentage of improvement in learning was calculated as the change in latency from first to last day and analyzed by Wilcoxon rank sum test. In the probe tests, latency to the first entry into GQ or PZ was analyzed by Kruskal-Wallis test and Wilcoxon rank sum test. Number of entries and duration spent in each quadrant were analyzed by a RM-ANOVA with epoch and quadrant as factors.

#### Y-Maze

With shipment 4, the Y-maze task was included as an additional assessment of spatial learning using a continuous spontaneous alternation paradigm (Lalonde 2002). PND38 rats from G2 and G4 were placed in the center intersection of a black, plexiglass Y-maze with 3 equal arms (56 × 10 x25cm) and allowed to explore for 7 min. Video-captured images were analyzed for arm entries using Ethovision XT. Sequential reentries into an arm were excluded and percent alternation was calculated as the number of triads containing entries into all three arms/maximum possible alternations. Wilcoxon Rank Sum tests were used to analyze the number of entries and % alternation.

### Tissue Collection and Fluoride Analysis

Following completion of MWM testing, samples were collected from rats randomly selected from shipments 3 and 4. Blood was collected via a cardiac puncture from rats deeply anesthetized under CO_2_ and plasma separated by centrifugation in EDTA-free, heparin coated plasma tubes (# 367874, Becton Dickinson, Franklin Lakes, NJ). The brain and femur were excised, immediately frozen on dry ice, and stored at -80 °C. Cleaned femur samples (5–8 mg) were ashed (8 h; 590 °C), pulverized*,* and weighed. Brain samples were homogenized in 3-ml di-H_2_O. For PND25 assessment, the brain and femur samples were obtained from randomly selected unassigned male rats. Duplicate samples were analyzed using a modification of the hexamethyldisiloxane microdiffusion method of Taves and Neuman (1964) and detected with a fluoride ion-specific electrode and a pH/ISE meter. Urine was collected in metabolism cages (Techniplast) from individual rats (*n* = 10) in G2 and G4 between 9:00–13:00 h and frozen. No water was provided over this interval. Urine samples < 200 μl were excluded. A 100-μl aliquot of urine was analyzed for creatinine using a kinetic modification of the Jaffe procedure (Moore and Sharer 2017). The rate of change at 520/800 nm was determined using the Olympus AU400e clinical analyzer (Beckman-Coulter Irving, TX).

#### Tissue Collection, Staining, and Histological Analysis

The kidney, liver, and reproductive system organs were collected following CO_2_ euthanasia at the termination of behavioral testing from randomly selected adult rats (> PND80) in cohorts 3 and 4 of shipment 3 (*n* = 8–13). The liver and kidney were immersion fixed in 10% neutral buffered formalin. Testes and epididymis were immersion fixed 24 h in Davidson’s. Samples were trimmed, ethanol dehydrated, embedded in paraffin, and 5 μm sections cut and stained with Hematoxylin and eosin (H&E). Randomly selected rats from G2 and G4 (*n* = 6; cohort 4) were deeply anesthetized with Fatal-Plus (Vortech Pharmaceuticals, Ltd., Dearborn, MI), whole body perfused with saline followed by 4% paraformaldehyde/phosphate buffered saline, and post-fix overnight at 4 °C. Brains were excised, transected in the mid-sagittal plane, and processed for paraffin embedding. H&E stained sections from each hemisphere (8 μm) were selected to represent a consistent plane of cut containing the hippocampus (lateral 1.35–1.95 mm) and immunostained for astrocytes and microglia. Endogenous peroxidase activity was quenched with 3% H_2_O_2_ followed by heat-induced epitope retrieval (0.01 M citrate buffer pH 6.0; Biocare Medical, Concord, CA). Non-specific binding was blocked with avidin/biotin (Vector Labs, Burlingame, CA) and 10% normal goat serum (Jackson Immunoresearch, West Grove, PA). Sections were incubated with rabbit anti-cow glial fibrillary acidic protein (Dako GFAP; 1:7000; RT; 30 min; Agilent Technologies, Carpinteria, CA) then incubated with biotinylated goat anti-rabbit IgG (1:500; Vector-Labs) and detected with Vectastain Elite ABC R.T.U. (Vector Labs); 3,3-diaminobenzidine (DAB, Agilent Technologies). Microglia were identified with a rabbit polyclonal antibody to ionized calcium-binding adaptor molecule 1 (Iba-1, 1:600, 1 h, 24 °C; Wako Chemicals, Richmond, VA) following microwave antigen retrieval. Defined regions of interest (ROI) of the suprapyramidal blade of the dentate gryus and the CA1 pyramidal layer were evaluated. Brain sections were scanned under × 20 magnification (Aperio ScanScope T2 scanner, Aperio Technologies, Inc., Vista, CA) and viewed using Aperio ImageScope v.6.25.0.1117. Stained slides were assigned random numbers and blinded for evaluation.

### Thyroid Hormone Analysis

From cohort 1 in shipment 3, six rats (PND56) per group were randomly selected and blood collected via cardiac puncture under CO_2_ anesthesia. Serum triiodothyronine (T3) and thyroxine (T4) analyses were performed using I^125^ radioimmunoassays (MP biomedicals LLC; Costa Mesa CA) following manufacturer’s instructions. A 100-μl aliquot of serum was incubated with 1 ml of T3 tracer (37 °C; 60 min) and radioactivity determined (APEX gamma counter; ICN Microbiomedic Systems, Huntsville, AL). For T4 determination, samples and standards (2–20 ng/dl) were incubated with T4 tracer (RT; 60 min) and processed as described for T3. The percent trace level was calculated and ng/dl determined from standard curve as adjusted for non-specific binding. Serum samples were assayed for thyroid stimulating hormone (TSH) (Rat Pituitary Magnetic Bead Panel Kit (Cat. no. RPTMAG-86K); EMD Millipore Corporation, Billerica, MA) according to manufacturer’s protocol. Briefly, 25-μl aliquot (diluted 1:3) was incubated in 200 μl of assay buffer with TSH magnetic beads (25 μl) overnight at 4 °C. Samples were washed, incubated with 50-μl detection antibody (RT, 30 min) followed by 50 μl of streptavidin-phycoerythrin (RT; 30 min), washed, 100 μl of Sheath Fluid added (5 min, RT) then a specific spectral address detected by phycoerythrin immediately analyzed (Luminex xPONENT; Luminex Corp., Austin, TX) using a fluorescent bead-based multiplexing system (LiquiChip-200; QIAGEN, Valencia, CA).

### Statistical Analysis

While fluorine is considered an essential element in human diet, how this applies to rodents is not known; however, lowering F^−^ levels in the specialized low-F^−^ chow to approximately 3 ppm had no impact on gestational outcome, eye-opening, and body weight. This allowed us to make comparisons across the drinking water dose groups maintained on the vehicle low-F^−^ chow. The experimental design for statistical analysis adhered to the following. Differences between G1 (control chow) and G2 (low-F^−^ chow) rats receiving RO-H_2_O for behavioral endpoints were assessed to confirm that the lower level of fluoride in the diet did not alter the normal expected pattern of behavior. Differences between G2 and G3 (10 ppm F^−^) or G4 (20 ppm F^−^) were tested to evaluate effects of fluoride in the drinking water on rats maintained on the low-F^−^ chow. Data were tested for homogeneity of variance using Levene’s tests and for non-normality using Shapiro-Wilk tests. Statistical significance was set at two-tailed *p* < 0.05. Group sizes were statistically determined as sufficient for detecting significant differences from controls at *p* < 0.05. In the absence of a significant interaction between shipment and exposure, shipment was not included as a factor in the final analysis. Analyses of F^−^ levels and levels of T3, T4, and TSH were conducted with Wilcoxon rank sum tests (G1 vs. G2) and Kruskal Wallis tests (G2, 3, and 4). Post-hoc comparisons were conducted with Dunn’s multiple comparisons tests. Specific analyses conducted for behavioral tests are reported in relevant methods sections. Statistical analyses were performed using GraphPad Prism 7 (GraphPad Software, Inc., La Jolla, CA); SAS 9.37 (SAS Institute, Cary, NC), and R (R Core Team 2016).

## Results

### Behavioral Assessments

#### RW Activity

All rats displayed a normal diurnal cycle with increased activity during the dark phase (dark areas) compared to light phase (Fig. 1a, b). A linear mixed-effects RM-ANOVA for daily dark or light phase rotations over 2 days showed similar activity across groups G1 and G2, and G3 (10 ppm F^−^; Fig. 1a). From shipment 4, G2 and G4 rats were evaluated on the RW for an extended 5-day period with no significant differences observed across groups (Fig. 1b).

#### Locomotor Activity

**Fig. 2:**
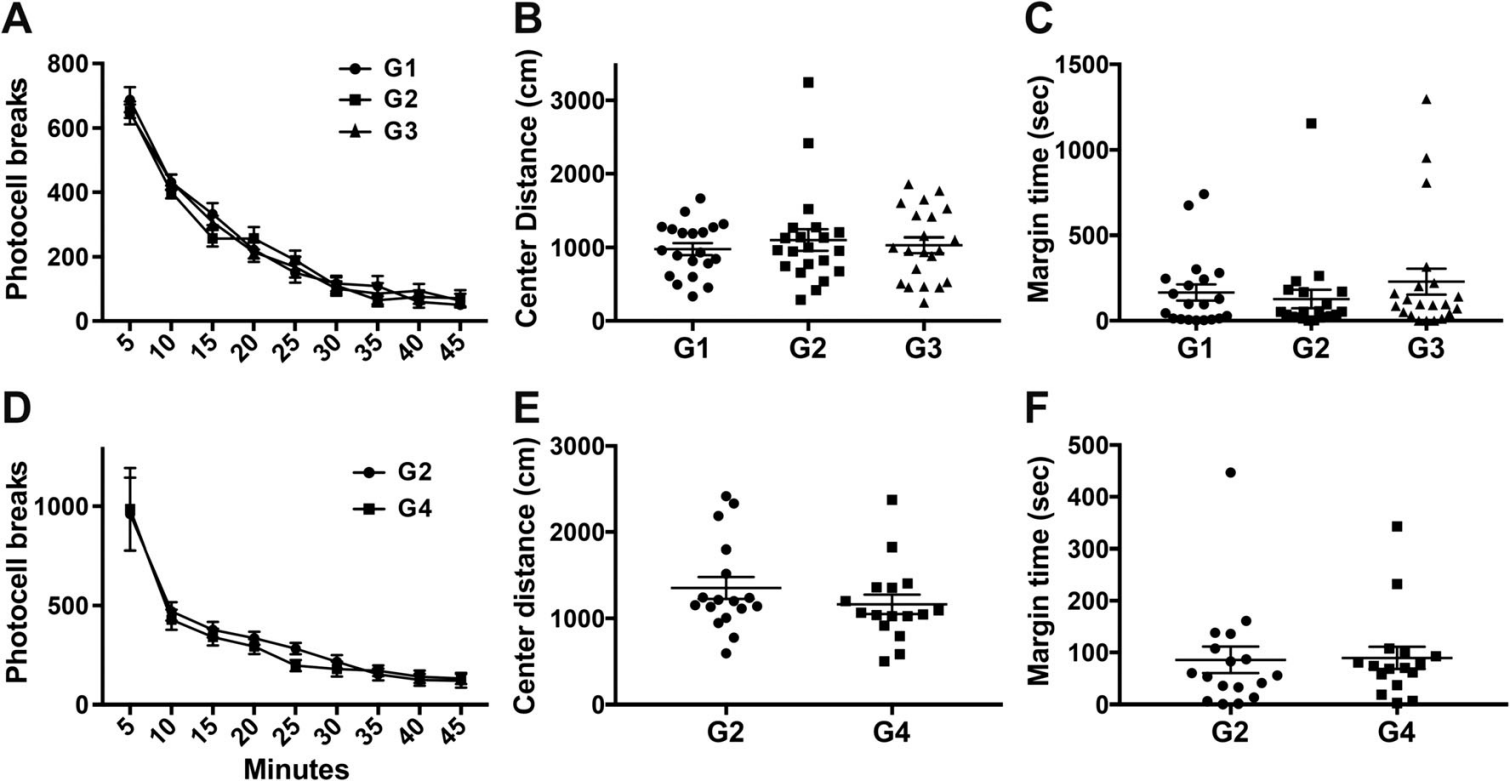
Open-Field activity in postnatal day (PND) 40 Long-Evans hooded male rats. (**a**) Ambulatory activity in open field in 5-min epochs (data represents mean ± SEM). (**b**) Total distance (cm) traveled in the center of the arena and (**c**) total time spent in the margin of the arena over 45-min session (data represents mean ± SEM and individual animal response). G1: standard chow/ RO-H_2_O (*n* = 20); G2: low-F^−^ chow/RO-H_2_O (*n* = 21); G3: low-F^−^ chow/10 ppm F^−^ drinking water (*n* = 21). (**d**) Ambulatory activity, (**e**) distance traveled in center, and (**f**) margin time for G2 (*n* = 17) and G4: low-F^−^ chow/20 ppm F^−^ drinking water (*n* = 16). Drinking water exposure began on gestational day 6

All rats showed a decrease in ambulatory activity over time indicative of acclimation to the novel environment (Fig. 2a, d). There was no evidence of an alteration in the normal exploratory activity in a novel environment and no statistically significant differences observed across groups in center arena activity (Fig. 2b, e) or time spent in margin (Fig. 2c, f).

#### Elevated Plus Maze

The elevated plus maze utilizes the natural conflict of a rodent under bright illumination conditions to avoid an open area for fear of danger versus exploration of a novel environment. Young behaviorally naïve rats showed no differences between groups for the number of entries into the open arm and the percentage of the total session spent in the open arms (Table 1). Prior experience to behavioral assessment on the RW did not alter EPM performance and no differences were observed between groups (Table 1). In adult rats, an overall increase in exploratory activity was observed with higher number of entries and percent time spent in the open arms than that seen in young rats. No differences were observed across groups (Table 1). With the modification of the EPM paradigm to include a “predatory threat,” rats showed decreased time spent in the open arms. No differences were observed between groups. For approximately 90% of the session, rats remained within the enclosed arms, entering into the open arms less than two times. No evidence of freezing behavior was detected.

**Table 1:** Elevated plus maze performance of Long-Evans hooded rats following drinking water exposure to fluoride

	PND 30 naive			PND 30 RW			Adult		
	G1	G2	G3	G1	G2	G3	G1	G2	G3
	(*n* = 18)	(*n* = 20)	(10 ppm) (*n* = 23)	(*n* = 19)	(*n* = 21)	(10 ppm) (*n* = 22)	(*n* = 19)	(*n* = 19)	(10 ppm) (*n* = 22)
Open arm entries	3.6 ± 0.6	3.0 ± 0.7	3.5 ± 0.6	4.4 ± 0.9	4.7 ± 0.6	3.5 ± 0.5	8.2 ± 1.1	8.6 ± 1.5	8.7 ± 1.5
Open arm duration %	8.2 ± 1.9	9.1 ± 2.5	12.3 ± 2.6	8.2 ± 1.6	11.1 ± 2.2	9.8 ± 1.6	16.9 ± 2.2	14.9 ± 1.8	16.6 ± 1.8
Closed arm entries	7.0 ± 0.8	7.4 ± 0.7	7.2 ± 1.0	10.1 ± 1.2	7.8 ± 0.8	8.9 ± 1.1	17.7 ± 1.4	15.4 ± 1.7	16.1 ± 2.2
	PND 30 predator			PND 30 RW			Adult		
	G2	G4		G2	G4		G2	G4	
	(*n* = 11)	(20 ppm) (*n* = 10)		(*n* = 17)	(20 ppm) (*n* = 19)		(*n* = 16)	(20 ppm) (*n* = 16)	
Open arm entries	0.6 ± 0.3	0.7 ± 0.3		3.4 ± 0.6	2.5 ± 0.5		8.4 ± 1.8	9.9 ± 1.5	
Open arm duration %	0.5 ± 0.4	1.1 ± 0.6		8.3 ± 1.9	6.3 ± 1.8		9.7 ± 1.7	10.6	
Closed arm entries	6.6 ± 0.9	6.1 ± 0.7		9.1 ± 0.9	7.4 ± 0.9		18.1 ± 2.3	19.4 ± 2.0	

Data represents mean ± SEM

Rats were exposed from gestational day 6

G1: standard chow (Teklad 2918)/RO-H_2_0

G2: low-F^−^chow (Teklad TD.160173)/RO-H_2_0

G3: low-F^−^chow (Teklad TD.160173)/10 ppm F^−^drinking water

G4: low-F^−^chow (Teklad TD.160173)/20 ppm F^−^drinking water

#### Light/Dark Place Preference

**Fig. 3:**
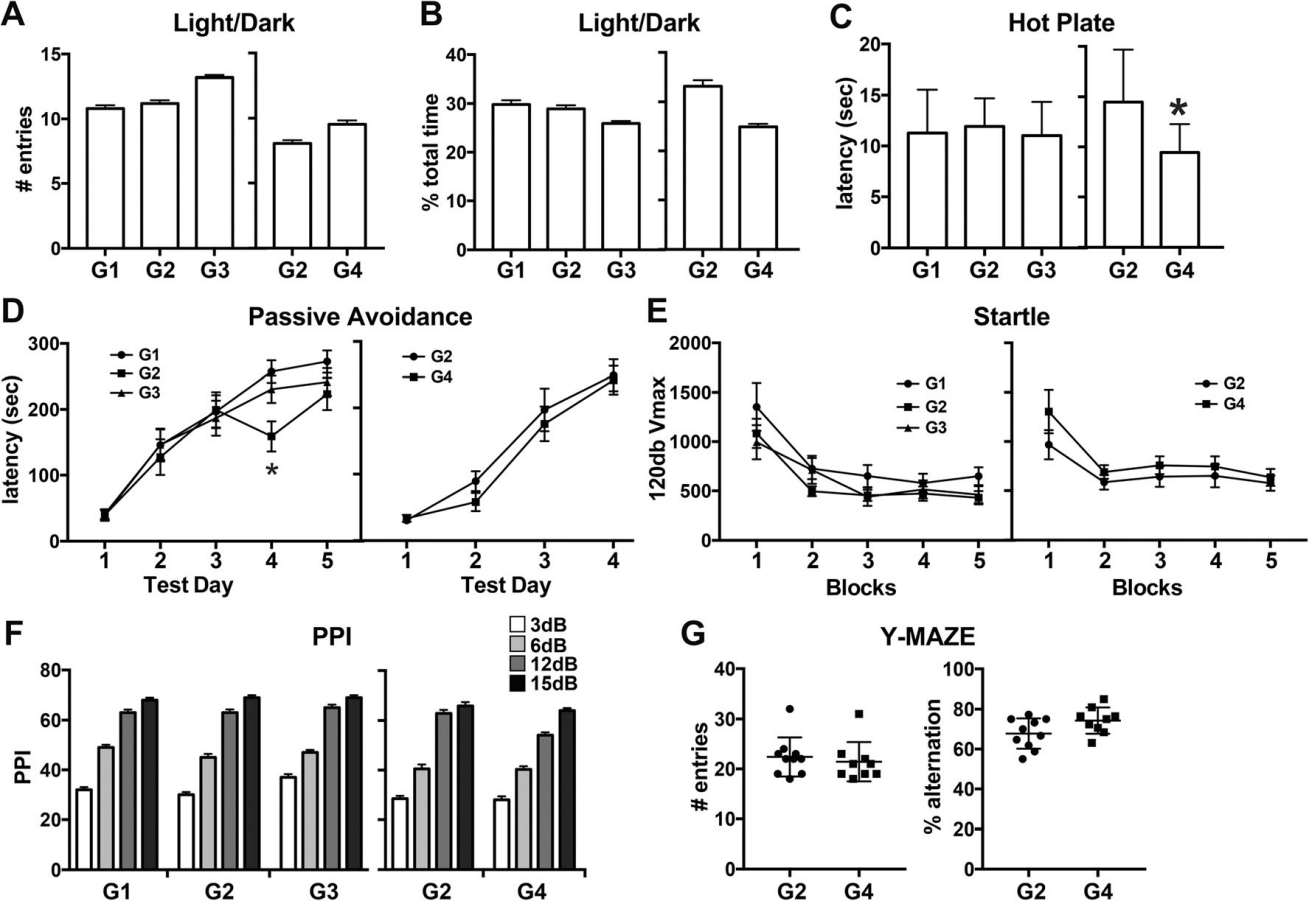
Light/dark place preference, hot-plate latency, passive avoidance, startle response, pre-pulse startle inhibition, Y-maze in Long-Evans hooded male rats. (**a**–**b**) Light/dark place preference at postnatal day (PND) 42. (**a**) Number of entries and (**b**) percent total time spend in lighted side. G1: standard chow/RO-H_2_O (*n* = 20); G2: low-F^−^ chow/RO-H_2_O (*n* = 21); G3: low-F^−^ chow/10 ppm F^−^ drinking water (*n* = 21) and G2 (*n* = 16) and G4: low-F^−^ chow/20 ppm F^−^ drinking water (*n* = 17). (**c**) Hot-plate latency at PND60 (G1: *n* = 13; G2: *n* = 13; G3: *n* = 14; and G2: *n* = 16, G4: *n* = 14). (**d**) Passive avoidance latency over days (G1: *n* = 13; G2: *n* = 13; G3: *n* = 14; and G2: *n* = 16, G4: *n* = 14). (**e**) 120 dB startle amplitude (Vmax) over blocks of 5 trials and (**f**) percent inhibition of 120 dB response from pre-pulse stimulus intensities of 3, 6, 12, or 15 dB above background. (PND 61–63; G1: *n* = 18, G2: *n* = 17; G3: *n* = 21; G2: *n* = 12, G4: *n* = 14). (**g**) Y-maze number of entries and percent alternation at PND60 from G2 (*n* = 10) and G4 (*n* = 9). Drinking water exposure began on gestational day 6. Data represent means ± SEM and individual animal responses. *p < 0.05

Examination of exploratory activity, reinforcement value of a dark chamber, and “anxiety” by light/dark place preference showed no differences between groups. The number of entries into the lighted side of the chamber (Fig. 3a) and the percent time spent within this chamber (Fig. 3b) showed no differences across groups in exploratory activity into the lighted side or preference for the dark chamber.

#### Hot Plate Latency

No differences in response latency to a 55 °C hot plate were observed between control groups and G3 (10 ppm F^−^). Control rats and rats exposed to 10 ppm showed no difference in latency. In rats exposed to 20 ppm F^−^ (G4), significantly shorter response latencies were observed (*p* < 0.002; Fig. 3c) indicative of hyperanalgesia.

#### Passive Avoidance

In all rats, latency to cross to the dark side was significantly increased (*p* < 0.01) over test sessions (Fig. 3d). No significant differences were observed across groups with a mean increase of between 190 and 230 s and a similar proportion of rats reached the maximum 300 s cutoff.

#### Startle Reactivity and PPI

Rats were assessed for auditory reflex response and sensory/motor gating. The first response to the novel auditory stimuli was not significantly different across groups. Habituation of the reflex response occurred over repeated trials, as evidenced by a progressive decrease in 120 dB Vmax, was similar for all groups (Fig. 3e). No differences were observed for Vmax recorded during no-stimulus trials. PPI was demonstrated for all rats and increased with increasing pre-pulse intensity levels (*p* < 0.0001; Fig. 3f). No group differences were observed for PPI at each pre-pulse intensity.

#### Y-Maze

To expand the evaluation of rats exposed to the higher dose level of 20 ppm F^−^, Y-maze performance was included to examine the willingness of rats to explore a new environment and the memory of the previously entered arm (Fig. 3g). No significant differences were observed across groups in the number of arm entries suggesting an equivalent level of exploratory activity. No significant differences were observed in across groups in memory of the previously entered arm and the preference for examining an alternate arm (percent alternation).

#### MWM

**Fig. 4:**
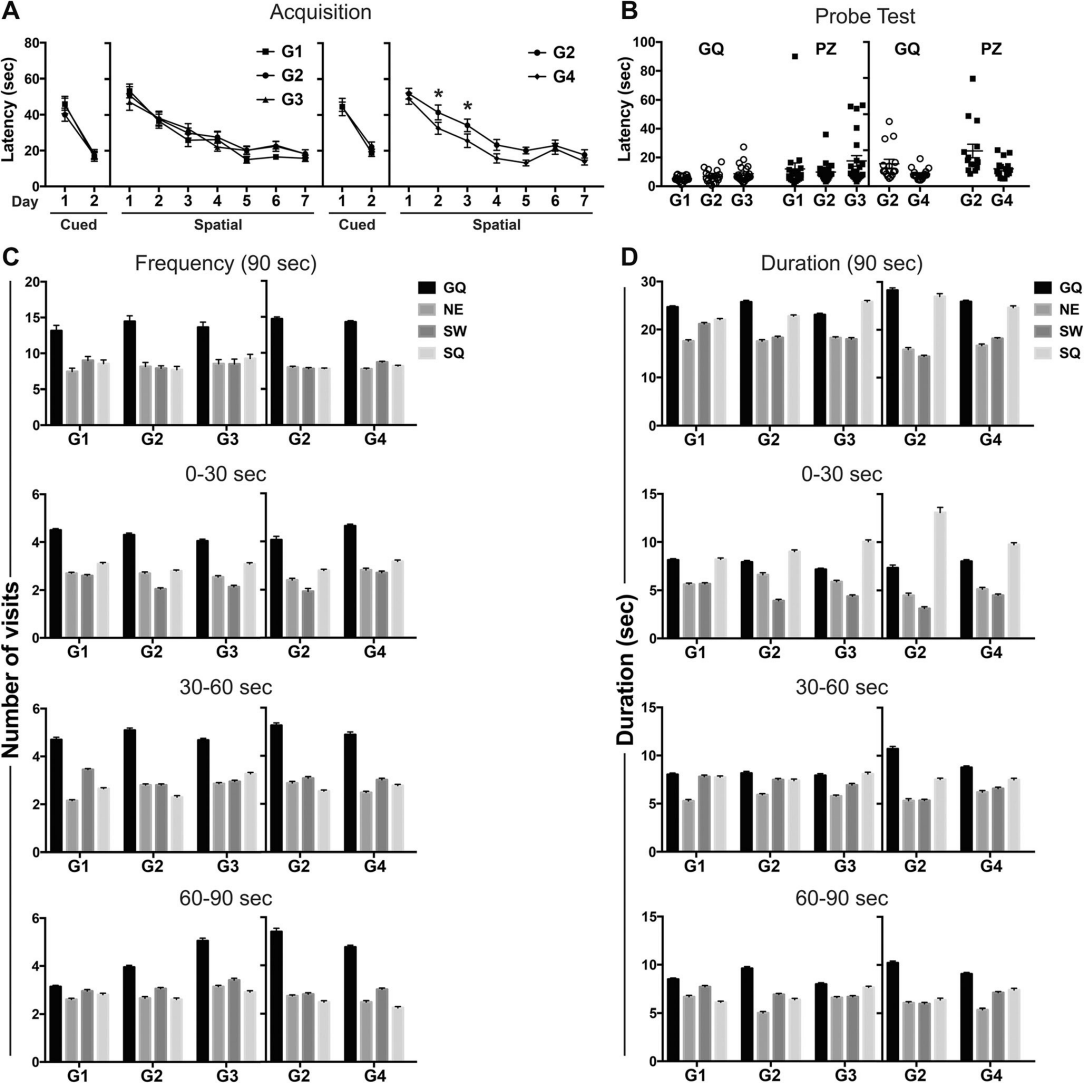
Morris water maze performance in adult Long-Evans hooded male rats. (**a**) Cued learning across 2 days and spatial training across 7 days. Data expressed as mean latency ± SEM (over three trials per day) to escape onto platform in the goal quadrant (GQ). A significant decrease in latency was observed over training (G1 vs. G2: F_(6,228)_ =  26.28; *p* < 0.0001; G2 vs. G3: F_(6,240)_ = 20.81; *p* < 0.0001; G2 vs. G4: F_(6,180)_ = 32.07; *p* < 0.0001). *significantly different (*p* < 0.01) as compared to G2. (**b**) Probe trial initial latencies to enter GQ or platform zone (PZ). (**c**–**d**) Probe trial distribution to quadrants across 90- and 30-s epochs for (**c**) # of visits and (**d**) duration of time in each quadrant. Data represents mean ± SEM and individual animal responses. G1: standard chow/ RO-H_2_O (*n* = 20); G2: low-F^−^ chow/RO-H_2_O (*n* = 20); G3: low-F^−^ chow/10 ppm F^−^ drinking water (*n* = 22) and G2 (*n* = 15) and G4: low-F^−^ chow/20 ppm F^−^ drinking water (*n* = 17). Drinking water exposure began on gestational day 6. Distinctions of the individual quadrants: GQ: Northwest goal quadrant; NE, Northeast; SW, Southwest; SQ, Southeast start quadrant

**Fig. 5:**
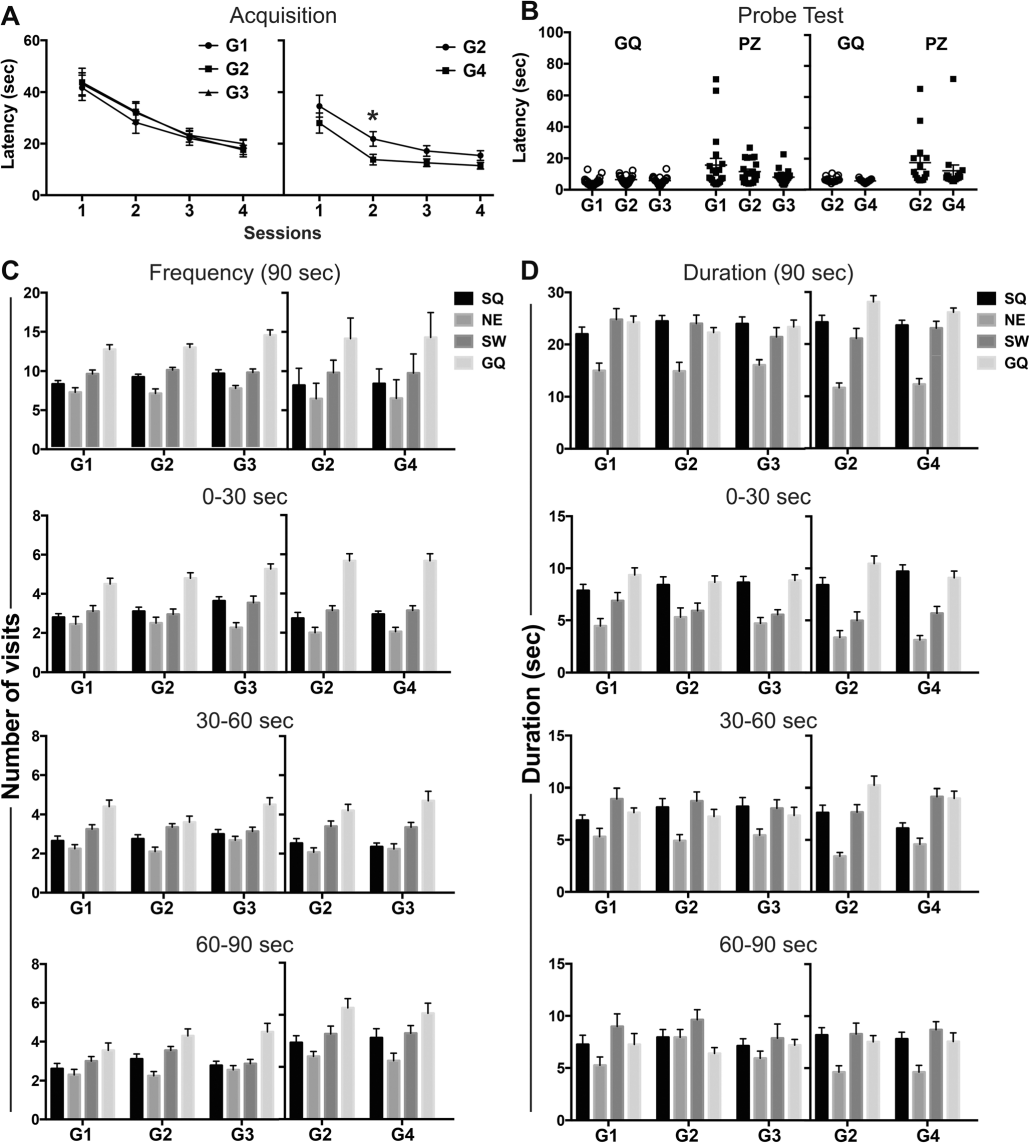
Morris water maze reversal learning of adult Long-Evans hooded male rats. (**a**) Acquisition as measured by mean latency (three trials per day) ± SEM to escape onto platform located in new goal quadrant (GQ). A significant decrease in latency was observed over training (G1 vs. G2: F_(3,114)_ =16.85; *p*<0.0001; G2 vs. G3: F_(3,120)_ =19.27; *p* <0.0001; G2 vs. G4: F_(3,90)_ =20.29; *p* <0.0001). *p < 0.01 as compared to G2. (**b**) Probe trial initial latencies to enter GQ or platform zone (PZ). Probe trial distribution to quadrants across 90- and 30-s epochs for (**c**) # visits and (**d**) duration of time in each quadrant. Data represents mean ± SEM. G1: standard chow/RO-H_2_O (*n* = 20); G2: low-F^−^ chow/RO-H_2_O (*n* = 20); G3: low-F^−^ chow/10 ppm F^−^ drinking water (*n* = 22) and G2 (*n* = 15) and G4 low-F^−^ chow/20 ppm F^−^ drinking water (*n* = 17). Drinking water exposure began on gestational day 6. Distinction of the individual quadrants: GQ, Southeast goal quadrant; SW, Southwest; NE, Northeast; SQ, Northwest start quadrant

In the first trial of cued learning and the first trial of hidden platform acquisition, the latency for reaching the visible platform was similar across groups (Fig. 4a). Latency decreased on day 2 with no significant differences seen across groups. In hidden platform spatial training, latency on the first day was not significantly different across groups (Fig. 4a) demonstrating similar initial performance. Learning was demonstrated by progressively shorter latencies over sessions (i.e., acquisition) in all groups (Fig. 4a). Overall, G4 rats exposed to 20 ppm F^−^ showed significantly shorter latencies to find the hidden platform (F_(1,30)_ = 10.43; *p* < 0.005), statistically significant on days 2 and 3, as compared to controls (*p* < 0.05; Fig. 4a). “Blinded” video tracking verification identified three G2 rats that floated upon entry into the pool. When excluded from the analysis, G2 continued to show significantly longer latencies than G4 across the session (F_(1,27)_ = 8.77; *p* < 0.05). No significant difference in path length was observed between groups (Supplementary Fig. S2a) suggesting that the difference in latency was not related to a difference in path selection to the platform. Memory was assessed in the probe test as latency to reach the previous platform location (platform zone, PZ) and preference for the goal quadrant (GQ). No significant differences were observed across G1, G2, and G3 (10 ppm F^−^) in latencies to reach the GQ or PZ (Fig. 4b). Similar to hidden platform training, rats exposed to 20 ppm F^−^ (G4) showed shorter latencies to reach the GQ (*p* < 0.05) or PZ (*p* < 0.01; Fig. 4b). “Floating” was observed in the same three rats identified in the hidden platform training. If these were excluded from analysis, significant differences in latency were no longer evident for the GQ. Preference for the GQ was evaluated for the entire 90-s session and 30-s epochs. A clear preference for the GQ was observed with number of visits (Fig. 4c). A preference for GQ was not observed with time spent (Fig. 4d) with similar values seen for the GQ and start quadrant (SQ). To further examine learning capability and flexibility to shift and learn a new escape location, reversal learning trials were conducted. All rats displayed shorter latencies on the first day of reversal learning as compared to the first day of hidden platform training. All groups showed a significant decrease in latency over reversal learning sessions (*p* < 0.001; Fig. 5a). Rats exposed to 20 ppm F^−^ showed overall shorter latencies across the session (F_(1,30)_ = 5.56; *p* < 0.05) with post hoc analysis showing significantly shorter latencies on day 2 as compared to G2 (*p* < 0.05; Fig. 4a). Due to the shorter latencies observed, distance traveled was examined in G2 and G4. No differences were observed between groups (Supplementary Fig. S2b). On the probe trial, no significant differences were observed in the initial latency to reach the GQ or PZ (Fig. 5b), number of visits or time spent in GQ (Fig. 5c, d).

### Fluoride Levels

In 25-day-old rats (Table 2), differences were observed for F^−^ levels in the brain as a function of chow with significantly lower levels observed in the low-F^−^ chow control rats (G2) as compared to normal chow G1 (*p* < 0.001). The addition of fluoride to the drinking water significantly elevated F^−^ levels in the brain over those seen in G2 (*p* < 0.01). The approximately 2× increase in brain levels in G4 with exposure to 20 ppm F^−^ over levels in G3 10 ppm F^−^ failed to reach statistical significance. In the femur, levels G2 were lower than all other groups (*p* < 0.001). While elevated, femur levels in the 10 ppm F^−^ rats (G3) failed to reach statistical significance as compared to G2 (*p* = 0.061). Femur levels in the 20 ppm F^−^ group (G4) were significantly higher than G2 (*p* < 0.0001). In adult rats (Table 2), G2 had lower plasma F^−^ levels as compared to all other groups (*p* < 0.001). In the brain, 10 ppm F^−^ did not significantly elevate F^−^ levels over G2. Exposure to 20 ppm F^−^ resulted in significantly higher levels of F^−^ in the brain as compared to G2 (*p* < 0.02). Accumulation of F^−^ in the femur was lowest in G2 receiving low-F^−^chow as compared to G1 receiving standard chow (*p* < 0.001); G3 receiving 10 ppm F^−^ (G3; *p* < 0.05); or G4 receiving 20 ppm F^−^ (*p* < 0.0001). Urine levels were significantly elevated in G4 as compared to G2 (*p* < 0.001).

**Table 2:** Fluoride levels in Long-Evans hooded rats following drinking water exposure to fluoride

Age	Sample	G1	G2	G3 (10 ppm)	G4 (20 ppm)
Weanling	Brain (μg/g)	0.0253** ± 0.01 (*n* = 6)	0.00 ± 0.0 (*n* = 6)	0.0477** ± 0.01 (*n* = 6)	0.0811** ± 0.04 (*n* = 6)
	Femur (μg/g)	216.7** ± 4.8 (*n* = 6)	35.2 ± 1.2 (*n* = 6)	235.0^b^ ± 25.0 (*n* = 6)	379.8*** ± 54.6 (*n* = 6)
Adult	Plasma (μg/ml)	0.018*** ± 0.003 (*n* = 10)	0.001 ± 0.000 (*n* = 10)	0.036*** ± 0.010 (*n* = 8)	0.025*** ± 0.005 (*n* = 10)
	Urine (μg/ml)		0.59 ± 0.14 (*n* = 8)		4.99** ± 0.96 (*n* = 8)
	Brain (μg/g)	0.35 ± 0.14 (*n* = 10)	0.21 ± 0.08 (*n* = 10)	0.27 ± 0.11 (*n* = 8)	0.85* ± 0.24 (*n* = 10)
	Femur (μg/g)	541.6*** ± 13.6 (*n* = 10)	56.79^a^ ± 2.1 (*n* = 8)	681.2* ± 43.8 (*n* = 10)	993.4*** ± 95.2 (*n* = 10)

Data represents mean ± SEM

Rats were exposed from gestational day 6 to tissue collection

G1: standard chow (Teklad 2918)/RO-H_2_0

G2: low-F chow (Teklad TD.160173)/RO-H_2_0

G3: low-F chow (Teklad TD.160173)/10 ppm F^−^ drinking water

G4: low-F chow (Teklad TD.160173)/20 ppm F^−^ drinking water

^b^*p* = 0.06, *p < 0.05, **p < 0.01, ***p < 0.001 as compared to G2

^a^Two outliers excluded (276.8 and 849.0 μg/g)

#### T3, T4, and TSH Levels

In adult rats, no differences were observed across groups for serum T3 or T4 levels (Table 3). As compared to G1 rats maintained on a standard chow diet, TSH levels were significantly lower in G2 rats maintained on low-F^−^ chow (*p* < 0.001). No significant differences in TSH levels were observed across groups maintained on the low-F^−^ chow as a function of F^−^ in the drinking water (Table 3).

**Table 3:** T3, T4, and TSH levels in Long-Evans Hooded rats following drinking water exposure to fluoride

	G1	G2	G3 (10 ppm)	G4 (20 ppm)
T3 (ng/dl)	153.80 ± 5.74 (*n* = 6)	137.80 ± 6.49 (*n* = 6)	143.20 ± 8.43 (*n* = 6)	148.00 ± 4.82 (*n* = 6)
T4 (μg/dl)	6.86 ± 0.36 (*n* = 6)	7.03 ± 0.34 (*n* = 6)	6.35 ± 0.59 (*n* = 6)	6.70 ± 0.23 (*n* = 6)
TSH (ng/ml)	18.93** ± 5.70 (*n* = 6)	2.83 ± 0.44 (*n* = 6)	2.65 ± 0.53 (*n* = 6)	2.35 ± 0.61 (*n* = 6)

Data represents mean ± SEM

Rats were exposed from gestational day 6

G1: standard chow (Teklad 2918)/RO-H_2_0

G2: low-F^−^chow (Teklad TD.160173)/RO-H_2_0

G3: low-F^−^chow (Teklad TD.160173)/10 ppm F^−^ drinking water

G4: low-F^−^chow (Teklad TD.160173)/20 ppm F^−^ drinking water

**p < 0.01 as compared to G2

### Histological Examination of the Brain

**Fig. 6:**
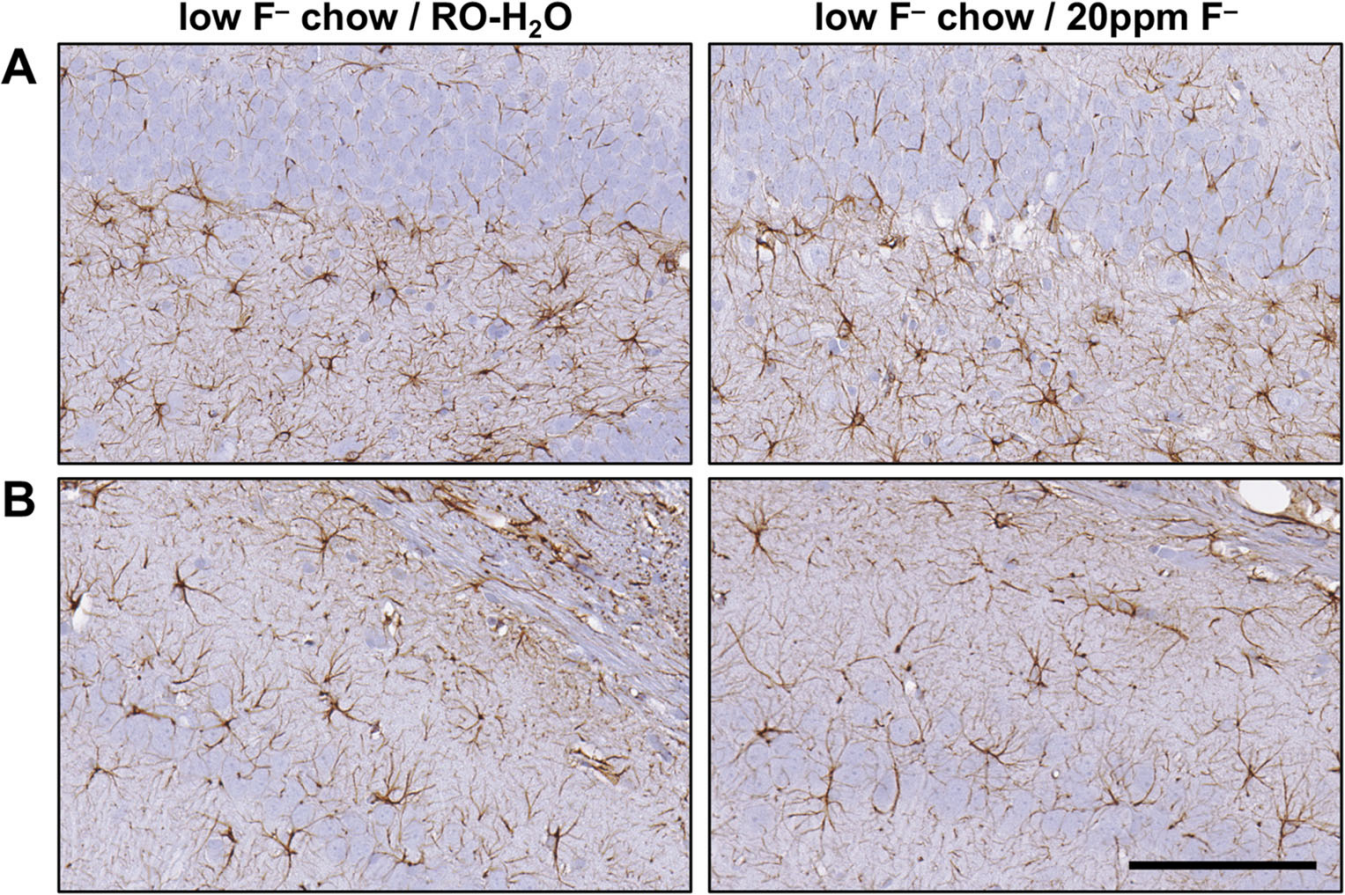
Representative images of GFAP+ astrocytes in the hippocampus of adult Long-Evans hooded male rats exposed to low-F^−^ chow/RO-H_2_O or low-F^−^ chow/20 ppm F^−^ drinking water beginning on gestational day 6. (**a**) Suprapyramidal blade of the dentate gyrus. (**b**) CA1 pyramidal cell layer. Cells displayed normal process-bearing morphology with no evidence of hypertrophy. 3,3-diaminobenzidine staining (brown). Hematoxylin counterstain (blue) showed no disruption of the normal morphology of the hippocampal regions and no evidence of neuronal death. (*n* = 6). Scale bar = 100 μm

**Fig. 7:**
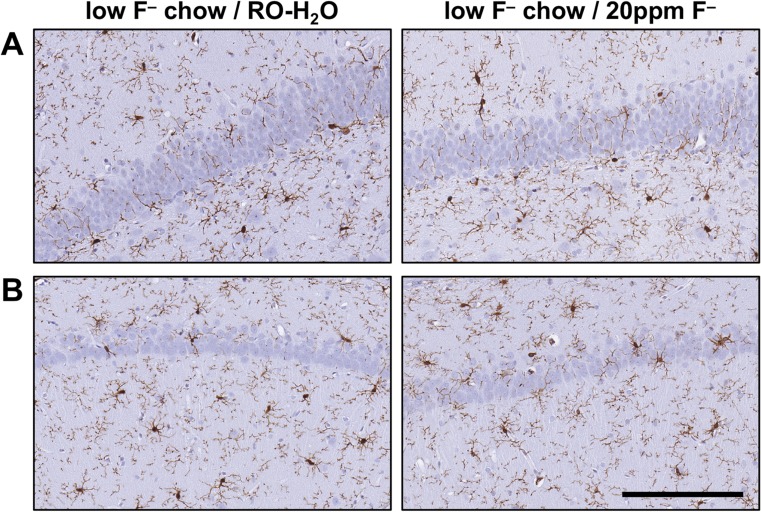
Representative images of Iba-1+ microglia in the hippocampus of adult Long-Evans hooded rats exposed to low-F^−^ chow/RO-H_2_O or low-F^−^ chow/20 ppm F^−^ drinking water beginning on gestational day 6. (**a**) Suprapyramidal blade of the dentate gyrus. (**b**) CA1 pyramidal cell layer. Cells displayed normal process-bearing morphology with no evidence of reactivity or activation. 3,3-diaminobenzidine staining (brown). Hematoxylin counterstain (blue) (*n* = 6). Scale bar = 100 μm

A survey of H&E, Nissl, and GFAP staining of the hippocampus did not detect evidence of neuronal death or morphological characteristics of glial reactivity normally associated with brain injury. A relatively uniform distribution of GFAP+ astrocytes was observed. There was no evidence of astrocyte hypertrophy in the suprapyramidal layer of the dentate gyrus and the CA1 pyramidal cell layer (Fig. 6). Iba-1 staining for microglia showed a relatively uniform distribution of process-bearing cells in the hippocampus with no evidence of hypertrophy or amoeboid morphology (Fig. 7).

### Pathology

**Fig. 8:**
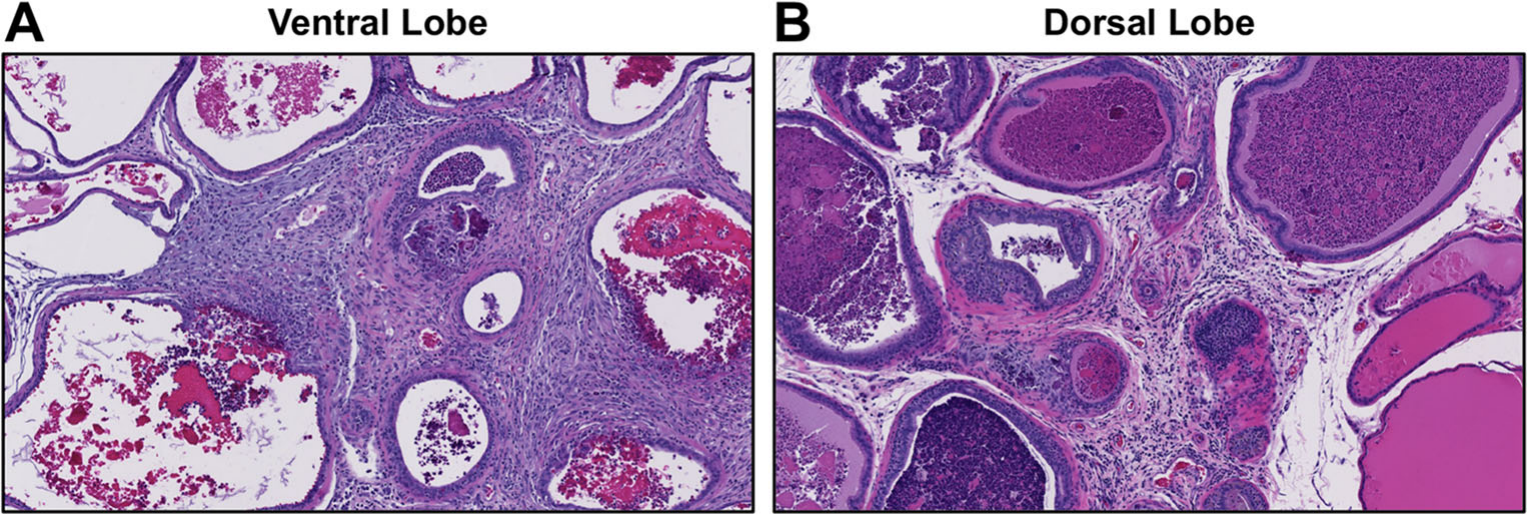
Representative images of prostate from adult male rats receiving to (G2) low-F^−^ chow/RO-H_2_O or (G4) low-F^−^ chow/RO-H_2_O 20 ppm F^−^ drinking water beginning on gestational day 6. Chronic inflammation is evident as dense infiltrating immune cells in the (**a**) ventral and (**b**) dorsal lobes with infiltration of mononuclear cells, expansion of interstitium, and concretions within the glandular lumen. Hematoxylin and eosin staining. A higher incidence of prostate inflammation was observed in G4 receiving low-F^−^ chow/20 ppm F^−^ (7 out of 13) as compared to G1 receiving standard chow/RO-H_2_O (0 out of 11), G2 receiving low-F^−^ chow/RO-H_2_O (2 out of 6), or G3 receiving low-F^−^ chow/ 10 ppm F^−^ (0 out of 8) drinking water

A general pathology examination was conducted on the heart, kidney, liver, testes, epididymides, prostate, and seminal vesicles (Supplementary Table S2). For the heart, kidney, liver, or testes, no differences were recorded for weight and no exposure-related pathology was observed. No F^−^-related changes were observed in the seminal vesicles or epididymides and there was no apparent decrease in the density or quantity of sperm within the epididymal tubules. In the 20 ppm F^−^ group, an increase in incidence (7/13 vs. 2/10) was observed for mild to moderate chronic inflammation in the prostate gland. Infiltration of mononuclear inflammatory cells within the interstitium was observed associated with concretions within affected glands (Fig. 8).

## Discussion

Male rat offspring maintained on a low-F^−^ chow and exposed to 10 or 20 ppm F^−^ in the drinking water beginning on GD6 showed a significantly elevated internal F^−^ burden in the weanling brain and femur as compared to levels observed in the low-F^−^ chow and normal drinking water. Deposition continued into adulthood. Drinking water exposure at these low levels was not found to alter motor performance or learning and memory in the test paradigms assessed. Low-level F^−^ did not alter thyroid hormone levels and produce neuronal damage or glia reactivity in the hippocampus, or histological damage in the heart, kidney, or liver. The low-level F^−^ diet did not show a significant effect on the behavioral endpoints examined. Effects observed at the 20 ppm F^−^ drinking water level included mild hyperanalgesia and an inflammatory response in the prostate.

Fluoride exposure occurs from multiple sources, yet the majority of studies reporting effects of fluoride on the nervous system were not often supported by analytical confirmation of exposure or consideration of contributions from drinking water source or chow. When reported, control water ranged from 0.15 to 1.77 ppm F^−^ and rodent chow between 10 and 25 ppm F^−^ (NTP 2016). Estimates of internal exposures have relied heavily on serum/plasma levels with limited studies providing data on urine excretion or deposition and accumulation in the bone or brain (Table 4; NTP 2016). Fluoride is rapidly absorbed from the gastrointestinal tract with transient peak plasma concentrations of approximate 30 min half-life. From the blood, F^−^ crosses the placenta (Shen and Taves 1974; Gui et al. 2010), less so into breast milk (Shen and Taves 1974; Ekstrand et al. 1981), and the blood-brain-barrier reduces entry into the brain (Whitford 1996). Calcified tissues such as the bone and teeth readily incorporate fluoride (NRC 2006) most prominently during periods of rapid growth and represent approximately 99% of the body burden of F^−^ that is not rapidly excreted by the urine (Kaminsky et al. 1990; Hamilton 1992; Whitford 1996). In humans, the average bone concentration is linearly related to water concentration and duration of exposure (Jackson and Weidmann 1958; Zipkin et al. 1958; Arnala et al. 1986; Rao et al. 1995). In rodents, F^−^ also accumulates in the bone (Rumiantsev et al. 1988; Bucher et al. 1991; de Carvalho et al. 2006; Gui et al. 2010; Dong et al. 2015a) increasing over age and exposure (Zipkin and McClure 1952; Ekstrand et al. 1994; Dunipace et al. 1995; Whitford 1999). In the current study, developmental exposure elevated F^−^ levels in the femur and brain at PND25 with continued deposition with age to levels comparable to previous studies (Table 4). A contribution from F^−^ in chow was also observed in the femur and brain and a differential absorption between water and food was demonstrated. With the standard chow, femur levels similar to that reported by de Carvalho et al. (2006) following 20 g F^−^/g chow but lower than levels observed with 20 ppm F^−^ in the drinking water.

**Table 4:** Summary of selected publications of fluoride levels in rats following fluoride exposure

Study	Rat strain	Age (*n*)	Fluoride (ppm)	Route	Duration	Brain [μg/g]^a^	Serum/plasma [μg/ml]^b^	Urine [μg/ml]^a^	Bone [μg/g]^b^
Adult exposure									
Balayssac et al. 2002^c^	SD	Adult (10 ♂)	0, 75, 150	DW	104 days		1, 5.5, 10.9 μm		
de Carvalho et al. 2006	Wistar	Adult (10♂)	0, 5, 15, 50	DW	120 days		0.03, 0.048, 0.115, 0.187		647, 808, 1414, 3439
Dong et al. 2015a	SD	Adult (30♂♀)	0.23, 22.6	DW	10 months			1.7, 2.16	1.52, 2.31
Ekambaram and Paul 2001	Wistar	Adult (8♀)	0, 226.24	DW	60 days		0.22, 1.94		
Gao et al. 2009a	SD	Adult (8 ♂♀)	0.23, 2.26, 22 6	DW	6 months			1.02, 2.59, 5.96	669.4, 1135.2, 1304.3
Gao et al. 2009b	SD	Adult (8 ♂♀)	0, 62.68	Gavage	3 months		0.018, 7.135	1.462, 64.966	
Jiang et al. 2014a	SD	Adult (8 ♂)	0, 54.3	DW	3 months	0.25, 1.19 CTX	0.09, 0.4		
Li et al. 2015	Wistar	Juvenile-adult (10 ♂)	0, 22.6	DW	3 months			6.67, 22.5	0.0646, 0.189
Lombarte et al. 2013	SD	Adult (10♀)	0, 15	DW	1 month		2.28,2.85		
Mullenix et al. 1995	SD	Adult (20 ♀)	0, 45.3	DW	6 weeks	0.406, 0.252 BG 0.358, 0.325 CB 0.479, 0.602 CTX 0.258, 0.79 HC 0.396, 0.308 HT 0.634, 0.306 MB	0.01, 0.077		
Rogalska et al. 2017	Wistar	Adult (8 ♂,♀)	0, 4.5, 22.6	DW	4 weeks		0.0541, 0.0596, 0.0823		
Rumiantsev et al. 1988^c^	Rat	Adult	0, 33.32	Gavage	30 days			14, 744 pg	2.3, 10.6 mg% thigh bone
Shalini and Sharma 2015	Wistar	Adult (10 ♂)	.9, 10	DW	2 months	0.55, 2.61	.048, .06		
Whitford et al. 2009	SD	Adult (8 ♀)	0, 33.2 85.6, 155.2	DW	8 months	0.082, 3.9, 6.42, 18.1 μmol/kg	0.32, 15.27, 25.72, 97.56 μmol/L		150, 5408, 9497, 14,847
Developmental exposure									
Banji et al. 2013	Wistar	Dam (6♀)	0, 20 mg/kg NaF	Gavage	GD6-PND11		Dams 0.12, 0.32 Pups 0.14, 0.42		
Basha et al. 2011	Wistar	Dam (8♀)	0, 100, 200	DW	GD0–3 months	0.318, 1.83, 2.32 CB 0.672, 2.28, 3.43 CC 0.258, 0.993, 1.93 HC 0.358, 2.12, 3.09 MO			
Dong Dong et al. 2015b	SD	Dams 10 months (30♀)	0, 50	DW	GD0-PND30 (10)			1.7, 2.16	35, 211
Gui et al. 2010^c^	SD	Dams 6 months (6♀)	6.2, 104 mg/kg NaF	Food	GD0-PND30 (10)	6.2 and 104 mg/kg NaF		0.98, 8.52	1124, 1873

^a^Values represent total F^−^ pg/g (ppm) unless otherwise noted

^b^Values represent total F^−^ pg/ml (ppm) unless otherwise noted

^c^Values presented as in publication

*DW*, drinking water; *BG*, basal ganglia; *CC*, cerebral cortex; *CB*, cerebellum; *CTX*, cortex; *HC*, hippocampus; *HT*, hypothalamus; *MB*, midbrain; *MO*, medulla oblongata

Many studies that examined effects of F^−^ exposure on learning and memory have relied on avoidance conditioning or maze performance. Such assessments can be influenced by activity level or motor deficits thus, requiring testing paradigms to address such confounders. Using an operant conditioning paradigm not dependent upon motor activity, Whitford et al. (2009) reported no learning deficit in rats exposed to F^−^ (10–50 ppm). Previous studies using a step-down avoidance procedure reported a deficit in rodents exposed to F^−^ (45–50 ppm) (Wang et al. 2004; Wu et al. 2006). In the current study, performance on a similar step-through passive avoidance task identified no exposure-related differences in learning. No differences were observed in light/dark place preference or pain perception that could have influenced PA performance. A slight thermal hyperanalgesia was observed in rats exposed to 20 ppm F^−^. This is consistent with thermal hyperalgesia and mechanical allodynia in rats exposed to 75 ppm F^−^ reported by Balayssac et al. (2002) but not with hypoanalgesia observed in rats exposed to 25 ppm F^−^ (Wu et al. 2008). As early as 1967, Elliott reported an absence of effect on maze performance following 4 weeks of exposure to 42.4 ppm F^−^. Using a food-reinforced maze task, Shalini and Sharma (2015) found no differences in errors but reported a longer latency on the first the first daily training trials over the first 2 weeks as a learning deficit in rats exposed to 10 ppm F^−^. The limited and transient nature of the response suggests a deficit in motor function rather than learning. Using different paradigms to assess learning and memory, the current study did not identify performance differences in rats exposed to ≤ 20 ppm F^−^ drinking water. No exposure-related differences were observed in Y-maze alternation rate, consistent with findings of Li et al. (2015). In studies using the MWM, a longer latency or greater distance traveled to the platform during the acquisition phase has been reported (Wu et al. 2008; Jiang et al. 2014b; Wei et al. 2014; Dong et al. 2015b). When acquisition data was reported, learning in exposed rats was demonstrated by a decrease in latency with training (Wu et al. 2008; Gui et al. 2010). In the few studies that included a MWM probe test, rats exposed to F^−^ in the chow (104 ppm; Gui et al. 2010) or drinking water (50 ppm; Dong et al. 2015b) showed deficits in isolated measures but preference for the escape quadrant was not reported. To address some of these limitations, the current study included recommendations of Vorhees and Williams (2006) and examined a number of aspects of the MWM performance including the sensitive measures of the number of visits and preference for goal quadrant over epochs. Using this approach, we did not observe differences in learning the MWM task, memory of the escape location, or ability to learn a new escape location.

In vitro studies have suggested a detrimental effect of fluoride exposure on neurons however, these effects occur at dose levels outside of the expected in vivo range of ppb (Table 4) (Zhang et al. 2008 (10–40 ppm); Lee et al. 2016 (5 mM NaF; 89 ppm F^−^); Inkielewicz-Stepniak et al. 2012; 1.9 and 4.75 ppm F^−^). For in vivo exposure, neuronal death or dysfunction was observed in the rat hippocampus (Basha et al. 2011; Shashi and Kumar 2016; Shivarajashankara et al. 2002; Yan et al. 2016; Teng et al. 2017) and neuronal shrinkage in the frontal cortex (Akinrinade et al. 2015). In the current study, hippocampal neurons displayed a normal morphology with no evidence of neuronal death. Neuronal death is normally accompanied by glia activation. Limited studies reported a stimulation of microlgia following F^−^ exposure (Shuhua et al. 2012; Yan et al. 2013, 2016). In the current study, consistent with the absence of neuronal death, astrocytes and microglia retained a normal process-bearing morphology and showed no evidence of hypertrophy or activation. There are a number of experimental factors that could account for the differences in neuropathology findings between studies including differences in F^−^ levels in chow and water source, as well as, potential processing artifacts for neuronal death (e.g., dark neurons, Zsombok et al. 2005; Jortner 2006).

At high exposure levels, fluoride exposure results in adverse effects on health. How this may reflect a specificity of effect on the nervous system or translates to lower exposure levels remains an important question. In many reports in the literature, the limited assessment across functional modalities and the over reliance on single terminal measures of performance such as, latency in the absence of an assessment of learning via demonstration of acquisition, limit the ability to evaluate a specific effect of F^−^ on learning and memory. A direct comparison of our findings to those in the literature is limited primarily by a lack of information on such details as analytical data for exposure, water source, and rodent chow type/brand, as well as, limited endpoints and details of behavioral assessments. Addressing such issues in future studies will enhance the ability to compare findings across studies and allow for a better assessment of the neurotoxicity associated with fluoride as it relates to level of exposure.

## Electronic supplementary material

Fig. S1 (GIF 473 kb)

High Resolution Image (TIFF 1522 kb)

Fig. S2 (GIF 63 kb)

High Resolution Image (TIFF 1121 kb)

Table S1 (GIF 58 kb)

High Resolution Image (TIFF 814 kb)

Table S2 (DOCX 24.2 kb)
