# Supplementary figures and images for: An Evaluation of Neurotoxicity Following Fluoride Exposure from Gestational Through Adult Ages in Long-Evans Hooded Rats
Source: Neurotox Res. 2018 Feb 5;34(4):781–98. doi: 10.1007/s12640-018-9870-x (PMC6077107; doi:10.1007/s12640-018-9870-x)

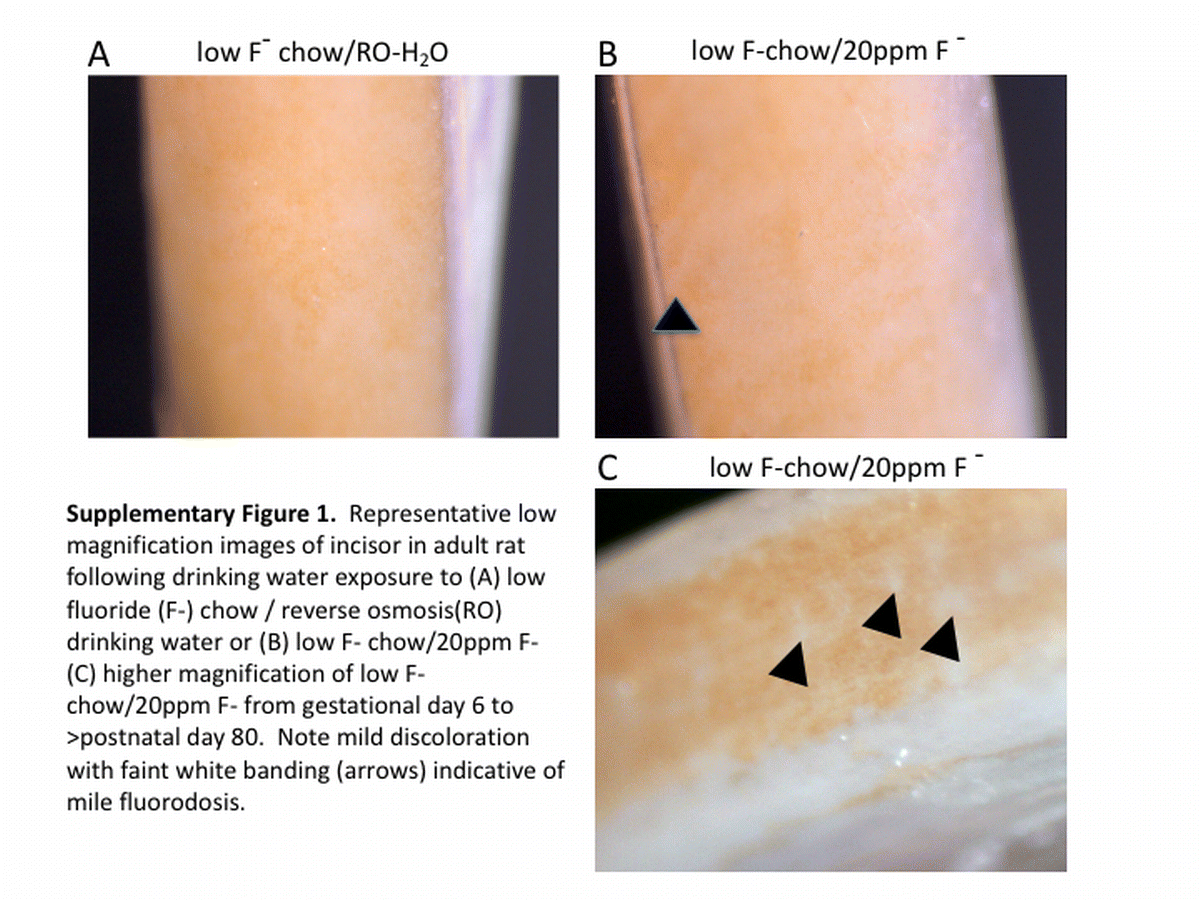

Supplement: Supplementary file 1 — (GIF 473 kb) [file 12640_2018_9870_Fig9_ESM.gif]

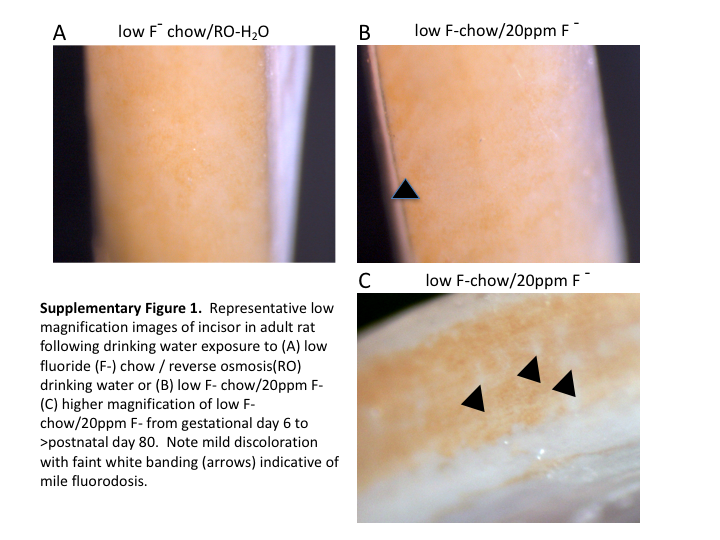

Supplement: Supplementary file 2 — High Resolution Image (TIFF 1522 kb) [file 12640_2018_9870_MOESM1_ESM.tiff]

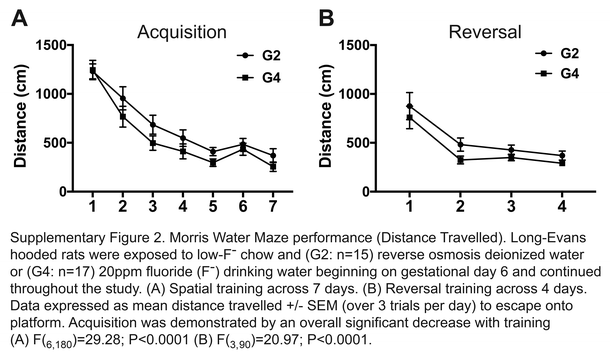

Supplement: Supplementary file 3 — (GIF 63 kb) [file 12640_2018_9870_Fig10_ESM.gif]

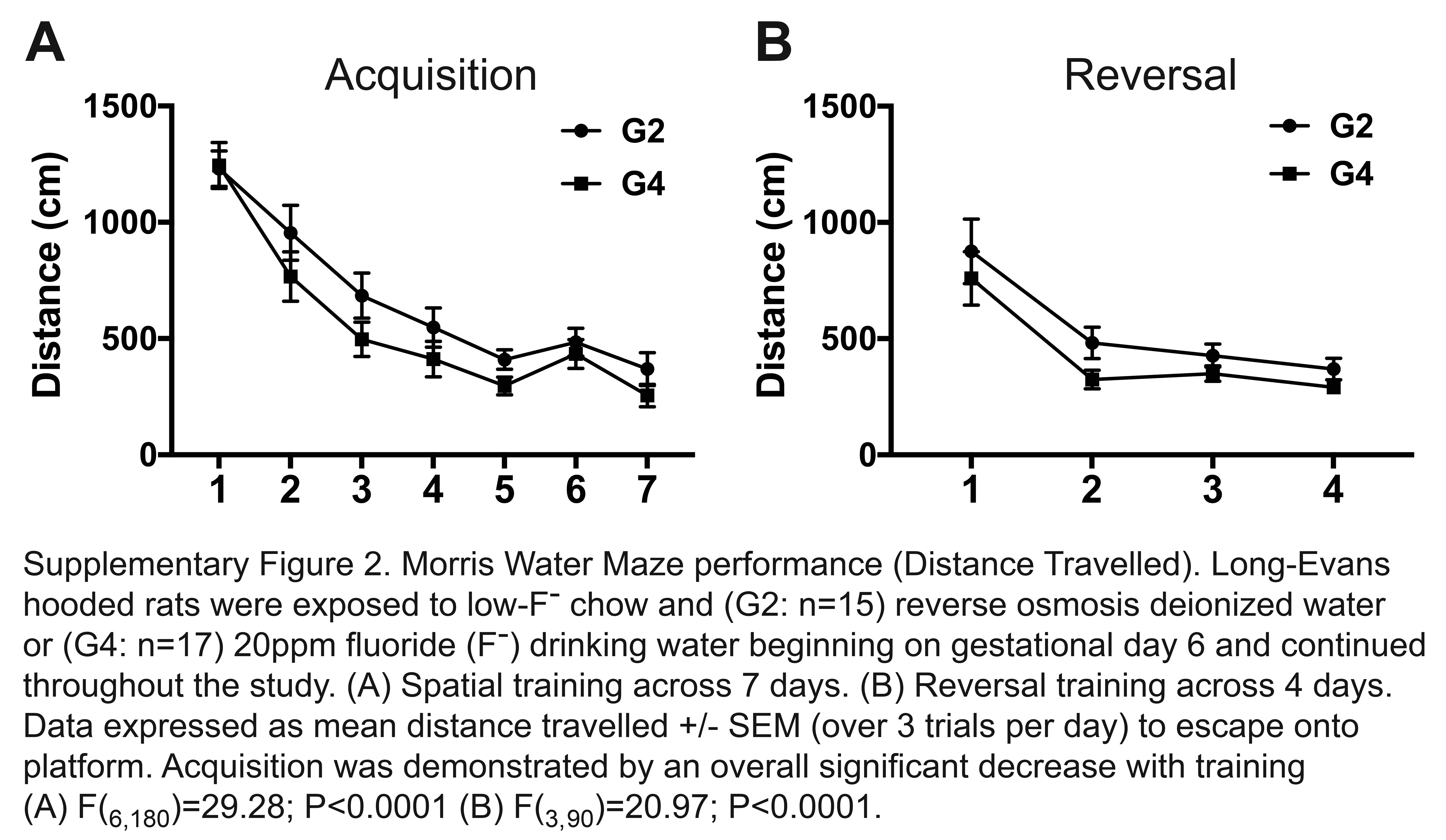

Supplement: Supplementary file 4 — High Resolution Image (TIFF 1121 kb) [file 12640_2018_9870_MOESM2_ESM.tif]

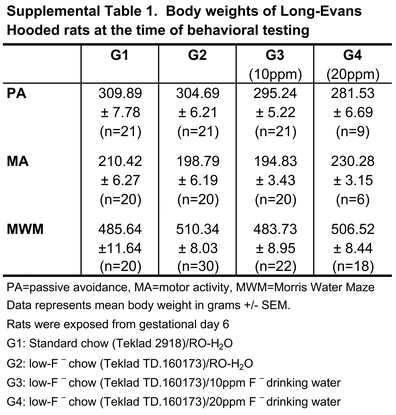

Supplement: Supplementary file 5 — (GIF 58 kb) [file 12640_2018_9870_Fig11_ESM.gif]

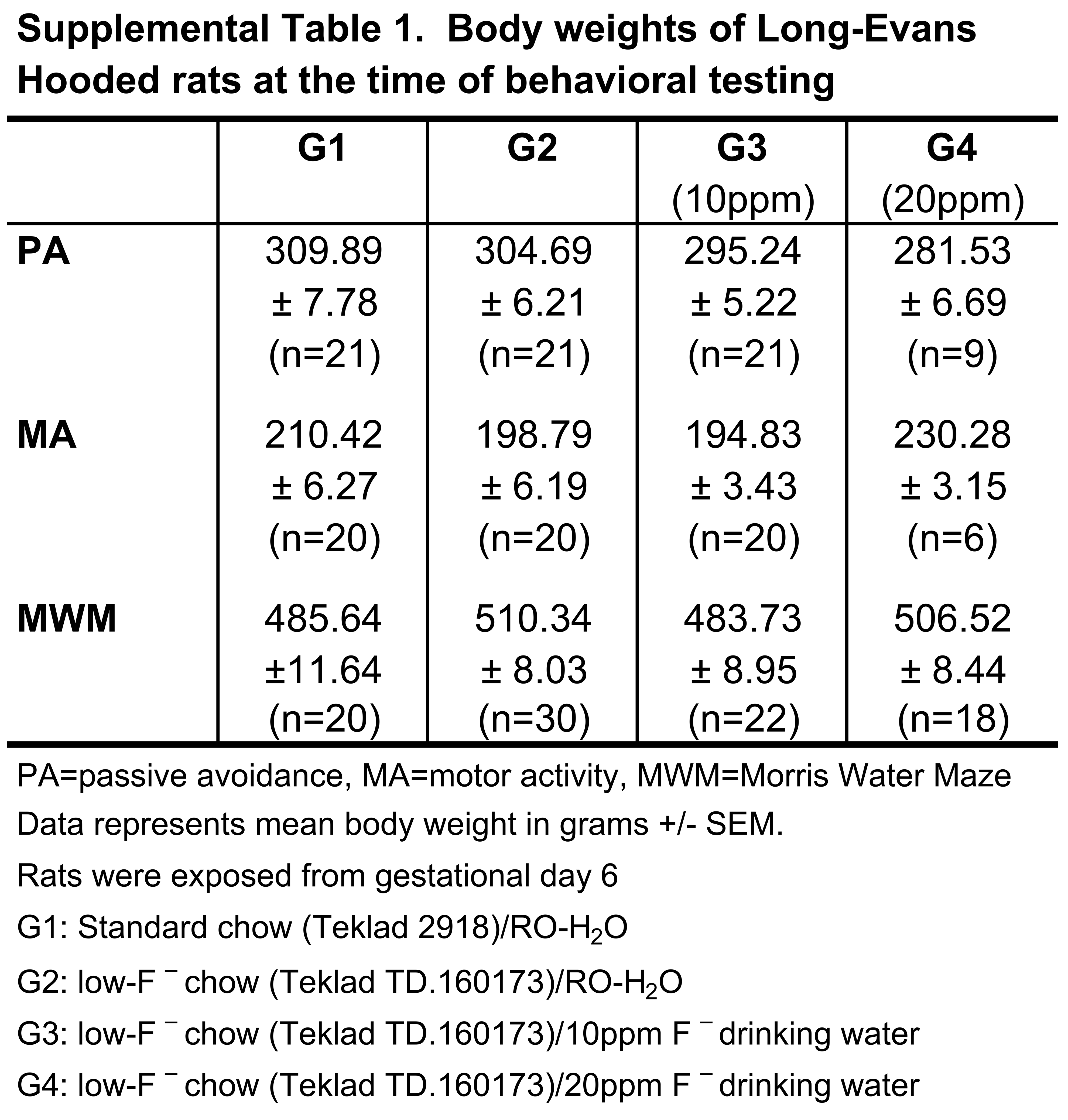

Supplement: Supplementary file 6 — High Resolution Image (TIFF 814 kb) [file 12640_2018_9870_MOESM3_ESM.tif]
