# Supplementary material for: An Evaluation of Neurotoxicity Following Fluoride Exposure from Gestational Through Adult Ages in Long-Evans Hooded Rats
Source: Neurotox Res. 2018 Feb 5;34(4):781–98. doi: 10.1007/s12640-018-9870-x (PMC6077107; doi:10.1007/s12640-018-9870-x)
Supplement: Supplementary file 7 — (DOCX 24.2 kb) [file 12640_2018_9870_MOESM4_ESM.docx]

**Supplementary Table 2: Histopathology of Tissue from Long-Evans Hooded Male Rats**

**Exposed to Fluoride in Drinking Water**

| **Tissue** | **G1** | **G2** | **G3** | **G4** |
| --- | --- | --- | --- | --- |
|  | **n  =  12** | **n=10** | **n  =  8** | **n  =  13** |
| **Testis  (No.  Examined)** | **12** | **10** | **8** | **13** |
| Degeneration,  tubular | 0 | 1 | 3 | 2 |
| *Minimal* | -­ | 1 | 2 | 0 |
| *Mild* | -­ | 0 | 1 | 1 |
| *Moderate* | -­ | 0 | 0 | 0 |
| *Marked* | -­ | 0 | 0 | 1 |
| Necrosis,  germ  cell   Minimal | 7 | 8 | 0 | 4 |
| Multinucleated  giant  cells,  Minimal | 0 | 0 | 0 | 1 |
| Exfoliation,  Minimal | 1 | 2 | 0 | 0 |
| Enlarged  residual  bodies,  Minimal | 1 | 2 | 0 | 1 |
| **Epididymides  (No.  Examined)** | **12** | **10** | **8** | **13** |
| Cell  Debris | 0 | 0 | 1 | 1 |
| *Minimal* | -­ | -­ | 1 | -­ |
| *Mild* | -­ | -­ | 0 | 1 |
| Hypospermia,  Marked | 0 | 0 | 0 | 1a |
| Cribriform  Change,  Moderate | 0 | 0 | 0 | 1a |
| **Seminal  Vesicles  (No.  Examined)** | **12** | **10** | **8** | **13** |
| Depletion | 4 | 1 | 2 | 4 |
| *Minimal* | -­ | -­ | 1 | -­ |
| *Mild* | 1 | 1 | 1 | 2 |
| *Moderate* | 3 | 0 | 0 | 2 |
| **Prostate  (No.  Examined)** | **11** | **6** | **8** | **13** |
| Inflammation,  chronic | 0 | 2 | 0 | 7 |
| *Mild* | -­ | 1 | -­ | 3 |
| *Moderate* | -­ | 1 | -­ | 4 |
| **Heart  (No.  Examined)** | **12** | **10** | **8** | **13** |
| Dilatation,  ventricles | 0 | 0 | 0 | 1 |
| **Kidney  (No.  Examined)** | **12** | **10** | **8** | **13** |
| chronic  progressive  nephropathy | 9  (1.1) | 6  (1.2) | 8  (1.6) | 11  (1.1) |
| hyaline  droplet  accumulation | 2  (1.0) | 2  (1.5) | 2  (1.0) | 5  (1.4) |
| pelvis,  dilation | 6  (2.3) | 3  (1.0) | 2  (2.5) | 4  (2) |
| mineralization | 5  (1.0) | 2  (2.0) | 5  (1.0) | 8  (1.0) |

| interstitium,  infiltration  cellular,   lymphocyte | 1  (1.0) | 0 | 0 | 0 |
| --- | --- | --- | --- | --- |
| Chronic  inflammation | 0 | 0 | 1  (4.0) | 0 |
| **Liver  (No.  Examined)** | **12** | **10** | **8** | **13** |
| chronic  active  inflammation | 12  (1.2) | 10  (1.3) | 8  (1.25) | 13  (1.2) |
| chronic  inflammation | 1  (1.0) | 1  (1.0) | 0 | 0 |
| fatty  change | 3  (1.0) | 6  (1.3) | 5  (1.2) | 5  (1.4) |
| basophilic  focus | 1 | 0 | 0 | 0 |
| capsule,  chronic  inflammation | 1  (1.0) | 0 | 0 | 0 |
| clear  cell  focus | 1 | 0 | 0 | 0 |
| necrosis,  focal | 0 | 0 | 0 | 1  (1.0) |

a  =  the  cribriform  change  and  hypospermia  were  in  same  epididymis  as  that  with  mild  cell  debris

Data  represents  incidence  (severity). G1 - standard chow/ RO-H2O drinking water, G2 - low-F^-^ chow/RO-H2O, G3 - low-F^-^ chow/10ppm F- ; G4 - low-F^-^ chow/20ppm F^-^ drinking water.
